# Kinetic modeling of phosphorylase-catalyzed iterative β-1,4-glycosylation for degree of polymerization-controlled synthesis of soluble cello-oligosaccharides

**DOI:** 10.1186/s13068-021-01982-2

**Published:** 2021-06-10

**Authors:** Mario Klimacek, Chao Zhong, Bernd Nidetzky

**Affiliations:** 1 Institute of Biotechnology and Biochemical Engineering, Graz University of Technology, NAWI Graz, Graz, Austria; 2 Austrian Centre of Industrial Biotechnology (Acib), Graz, Austria

**Keywords:** Cellodextrin phosphorylase (EC 2.4.1.49), Cello-oligosaccharides, Cellulose, Kinetic analysis and modeling, Iterative glycosylation, Bottom-up oligosaccharide synthesis

## Abstract

**Background:**

Cellodextrin phosphorylase (CdP; EC 2.4.1.49) catalyzes the iterative β-1,4-glycosylation of cellobiose using α-d-glucose 1-phosphate as the donor substrate. Cello-oligosaccharides (COS) with a degree of polymerization (DP) of up to 6 are soluble while those of larger DP self-assemble into solid cellulose material. The soluble COS have attracted considerable attention for their use as dietary fibers that offer a selective prebiotic function. An efficient synthesis of soluble COS requires good control over the DP of the products formed. A mathematical model of the iterative enzymatic glycosylation would be important to facilitate target-oriented process development.

**Results:**

A detailed time-course analysis of the formation of COS products from cellobiose (25 mM, 50 mM) and α-d-glucose 1-phosphate (10–100 mM) was performed using the CdP from *Clostridium cellulosi*. A mechanism-based, Michaelis–Menten type mathematical model was developed to describe the kinetics of the iterative enzymatic glycosylation of cellobiose. The mechanistic model was combined with an empirical description of the DP-dependent self-assembly of the COS into insoluble cellulose. The hybrid model thus obtained was used for kinetic parameter determination from time-course fits performed with constraints derived from initial rate data. The fitted hybrid model provided excellent description of the experimental dynamics of the COS in the DP range 3–6 and also accounted for the insoluble product formation. The hybrid model was suitable to disentangle the complex relationship between the process conditions used (i.e., substrate concentration, donor/acceptor ratio, reaction time) and the reaction output obtained (i.e., yield and composition of soluble COS). Model application to a window-of-operation analysis for the synthesis of soluble COS was demonstrated on the example of a COS mixture enriched in DP 4.

**Conclusions:**

The hybrid model of CdP-catalyzed iterative glycosylation is an important engineering tool to study and optimize the biocatalytic synthesis of soluble COS. The kinetic modeling approach used here can be of a general interest to be applied to other iteratively catalyzed enzymatic reactions of synthetic importance.

**Supplementary Information:**

The online version contains supplementary material available at 10.1186/s13068-021-01982-2.

## Background

Cellodextrin phosphorylase (CdP; EC 2.4.1.49) catalyzes the consecutive (non-processive) depolymerization of cello-oligosaccharides (COS) in the presence of phosphate, forming α-d-glucose 1-phosphate (αGlc1-*P*) as the cleavage product [1, 2]. The COS are β-1,4-linked gluco-oligosaccharides well-known for the fact that they are released during the hydrolysis of cellulose [3]. COS are soluble in water up to a degree of polymerization (DP) of about 6 [4, 5]. COS of higher DP self-assemble spontaneously in solution and thus precipitate as solid cellulose [6–8]. The CdP reaction is freely reversible, with the forward direction referred to as phosphorolysis and the reverse direction referred to as synthesis [1, 2, 9].

Due to mass action of phosphate present in excess over αGlc1-*P* in the cell, phosphorolysis is the preferred direction of reaction in vivo [10]. However, the CdP reaction in reverse direction under ex vivo conditions can provide an interesting route for the bottom-up synthesis of COS. Iterative β-1,4-glycosylation of cellobiose from αGlc1-*P* was shown in several studies [6–8, 11, 12] and it was recently demonstrated for the enzymatic production of COS at ~ 100 g/L final concentration [13]. The COS isolated from the process were shown to stimulate the growth of certain probiotic bacteria (e.g., *Clostridium butyricum*; *Lactococcus lactis* subsp. *lactis*), suggesting that they could be interesting dietary fibers providing a selective prebiotic effect [13]. Based on the emerging evidence on possible applications of COS for food and feed use [14], there is considerable interest in the intensification of the CdP-catalyzed conversion for the development of an efficient biocatalytic production.

The bottom-up synthesis of COS is kinetically complex, not only for the iterative glycosylation process that it involves, but also because precipitation-prone oligosaccharide products of DP ≥ 6 can be released in its course [6–8]. An efficient synthesis of soluble COS must therefore include control over the DP distribution of the oligosaccharide products formed, so as to avoid loss into insoluble material. It is intuitive, and has been demonstrated in previous studies, that the average DP of the COS depends on the molar ratio of cellobiose acceptor and αGlc1-*P* donor used in the reaction [6, 8, 15]. However, additional process parameters must be considered, in particular the substrate concentration and the reaction time, and there exists a complex relationship between these process parameters and the reaction output obtained (i.e., product yield, composition of the COS, insoluble portion).

We considered that a mathematical model describing the CdP-catalyzed build-up of the COS from cellobiose linked to the formation of insoluble material from the aggregation-prone portion of the COS would be fundamentally important to promote the enzymatic synthesis. Besides kinetic-mechanistic insight leading to advanced process understanding, the model could represent an important engineering tool for in silico window-of-operation analysis and reaction optimization. This would enable rational development of a biocatalytic process designed to have tailored performance characteristics. The approach worked out here for the CdP-catalyzed synthesis of COS could be of general importance for enzymatic iterative glycosylation applied to oligosaccharide synthesis [16–20]. Besides CdP, other glycoside phosphorylases [16–19] as well as trans-glycosidases [21] and sugar nucleotide-dependent glycosyltransferases [22, 23] are able to catalyze polymerization reactions and their corresponding products have drawn substantial interest across scientific disciplines and industrial sectors.

Development of a mechanism-based kinetic model for the CdP reaction was built upon well-established relationships between structure and function for the enzyme [24]. CdP is classified by sequence similarity as member of the glycoside hydrolase family GH94 [2, 9]. CdP uses a ternary complex kinetic mechanism in which both substrates must bind to the enzyme before the catalytic reaction happens [25–27]. The glycosyl transfer is a single-step catalytic process involving a stereo-chemically inverting, nucleophilic substitution at the anomeric carbon of the transferred glucosyl residue [2, 28]. The selectivity of CdP is strictly β-1,4 [29, 30]. A crystal structure of the CdP from *Clostridium thermocellum* in complex with cellotetraose reveals three subsites + 1 to + 3 for binding of the glucosyl/glucose residues of the acceptor substrate [27]. The catalytic subsite -1 accommodates the glucosyl residue of αGlc1-*P*. Substrate binding is ordered whereby αGlc1-*P* binds before the disaccharide or oligosaccharide acceptor [25–27]. It is relevant for the kinetic model of iterative β-1,4-glycosylation from cellobiose that enzyme subsites + 1 and + 2 show much stronger interaction with the bound sugar residues than subsite + 3 does [27]. Results of previous kinetic studies are in agreement with the structural evidence, showing that in terms of *k*_cat_ (turnover number) and *K*_M_ (apparent Michaelis constant), cellobiose is comparable an acceptor substrate for β-1,4-glycosylation from αGlc1-*P* as are soluble COS of DP 3 to 5 [25, 27, 31].

Here, we present a detailed time-course analysis of COS synthesis from cellobiose using the CdP from *C. cellulosi* (*Cc*CdP) [6] and develop a new Michaelis–Menten type mathematical model for the enzymatic conversion. We expand this model with an empirical description of the kinetics of the DP-dependent self-assembly of the COS into insoluble cellulose. A novel type of hybrid (empirical-mechanistic) model (for general case, see reference [32, 33]) is thus obtained for the enzymatic polymerization process. Based on kinetic parameters determined from time-course fits, this hybrid model was shown to give an excellent description of the experimental dynamics of the COS in the DP range 3–6 and also accounts well for the insoluble product formation. We demonstrate application of the model to establish conditions for COS production that maximize the soluble product concentration at minimal loss of the product to insoluble cellulose.

## Results and discussion

### Time-course analysis of the enzymatic COS synthesis

**Fig. 1:**
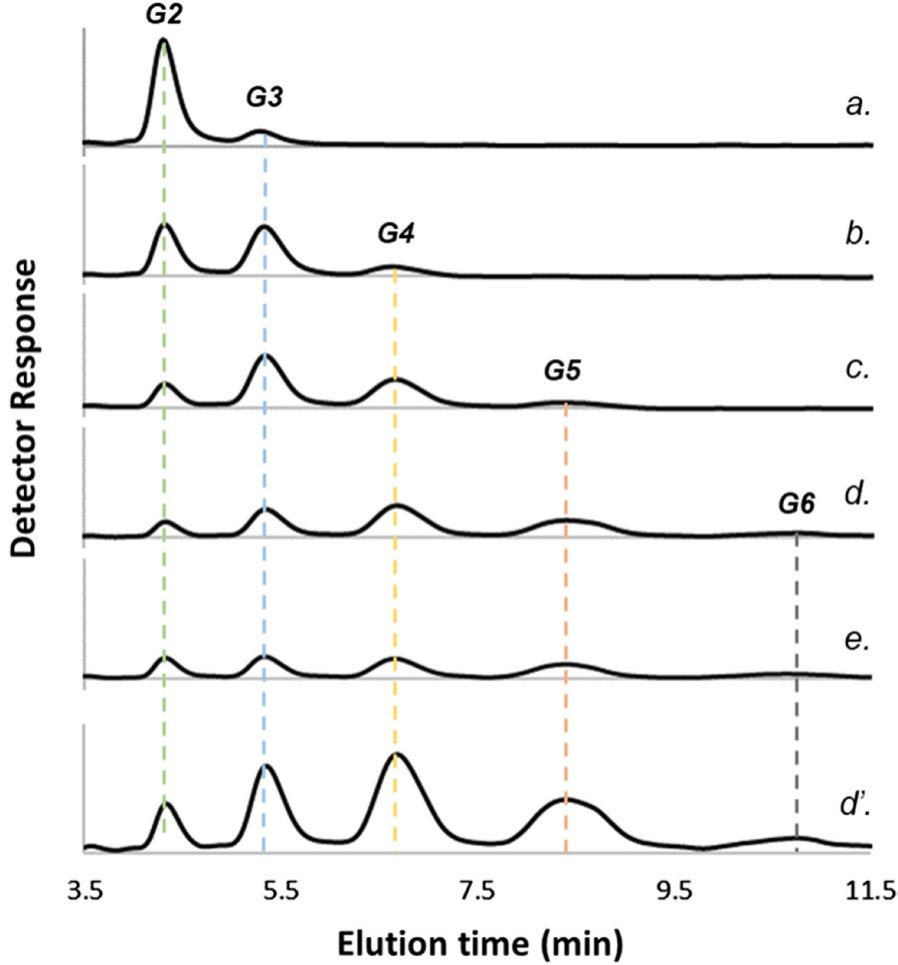
HPLC elution profiles of the selected reaction mixtures. Enzymatic reaction was conducted in the following conditions: 25 mM G2, 100 mM αGlc1-*P* (αGlc1-*P*/G2 ratio of 4.0), 1 U/mL *Cc*CdP in 50 mM MES buffer (pH 7.0), at 45 °C with reaction time of *a*, 5 min; *b*, 20 min; *c*, 40 min; *d*, 90 min; and *e*, 360 min. Analysis was done on a Hitachi LaChrom HPLC system equipped with a Luna 5 µm NH_2_ column (100 Å, 250 × 4.6 mm) operated at 40 °C. Acetonitrile–water (70:30, v/v) was used as eluent at a flow rate of 1.5 mL/min. Refractive index detection was used. Axis scale of detector response (y) was the same for *a-e*, and *d’* was the profile of *d* shown with smaller scale.

**Fig. 2:**
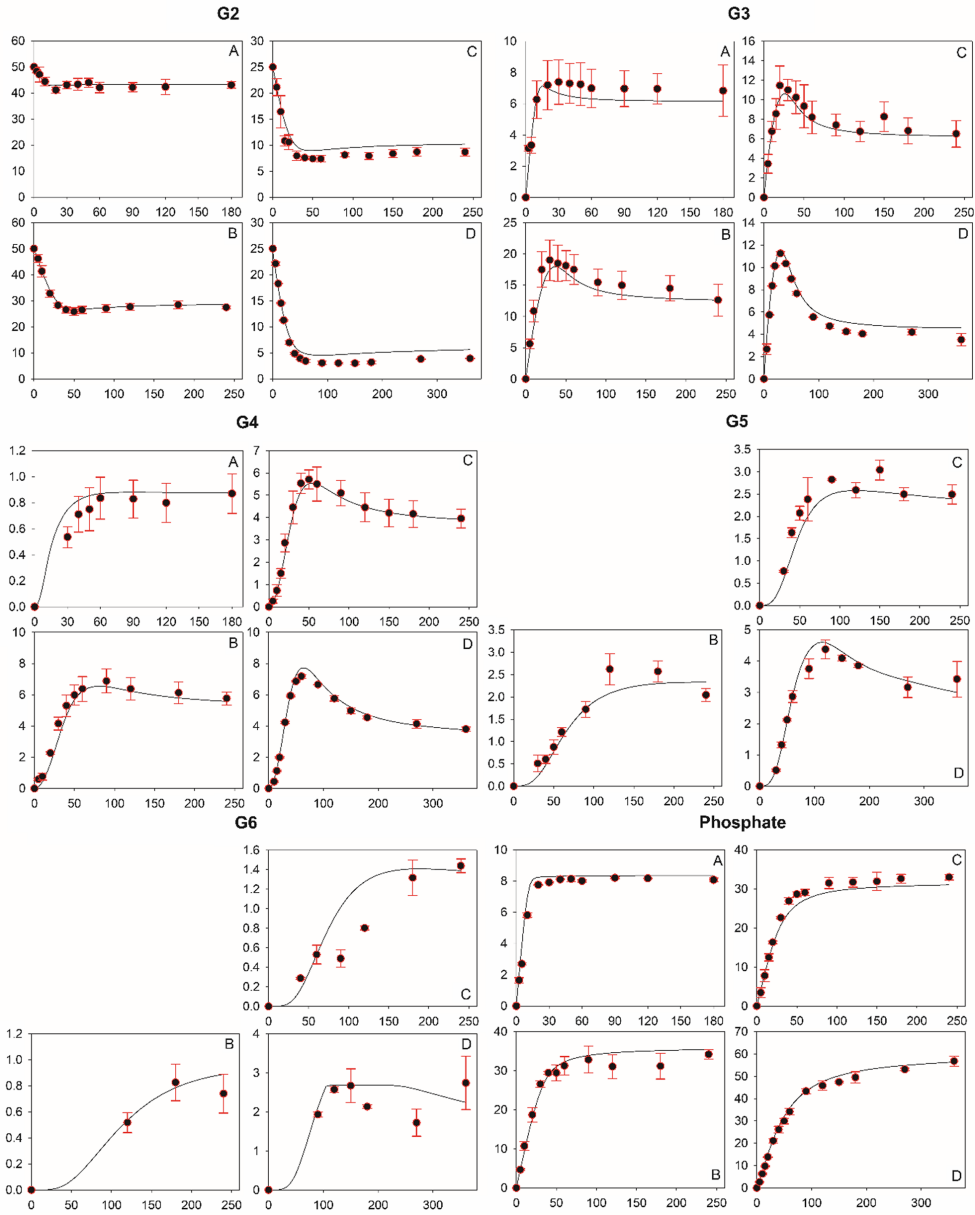
Results from experimental time-course analysis and parameter estimation by modeling. Experimental data are shown as black circles and standard deviations are indicated by red error bars. Fitted time courses (those from model PE18 are shown here) are presented as solid lines. **A**–**D** refer to experiments with αGlc1-*P*/G2 ratios of 0.2 (10 mM/50 mM), 1.0 (50 mM/50 mM), 2.0 (50 mM/25 mM), and 4.0 (100 mM/25 mM), respectively. x-axis and y-axis are represented by time in minutes and concentration in mM, respectively

The molar substrate ratio of αGlc1-*P* and cellobiose determines the DP distribution in the COS products released from the CdP reaction [6, 8, 15]. The larger this ratio, the greater is the abundance of high-DP products (DP ≥ 5) and so the portion of total product going into insoluble material. Synthesis reactions were performed at different αGlc1-*P*/cellobiose ratios (0.2–4.0) to represent situations of a variable extent of COS precipitation in our experimental data set. Using HPLC method [6], the COS of DP 2–6 were well separated for quantitative analysis, as illustrated in Fig. 1. The phosphate release was additionally measured. The experimental time courses are shown in Fig. 2. Overall, there was close mass balance between substrates consumed and products released in soluble and insoluble form. In the following, we identify cellobiose as G2 and the individual COS as Gn where n indicates the DP.

**Fig. 3:**
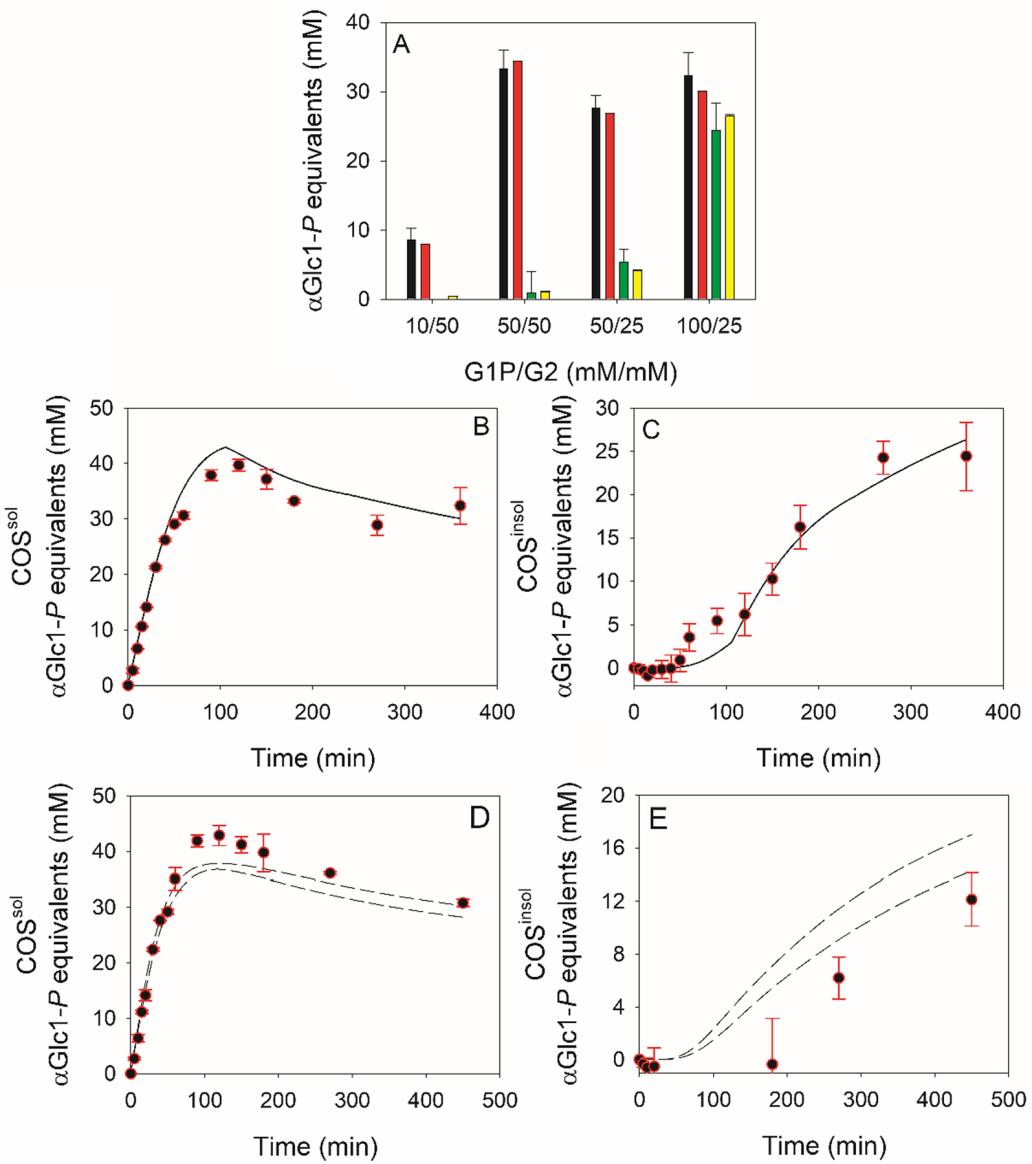
Soluble and insoluble COS pools expressed in the form of αGlc1-*P* equivalents. **A** Soluble (black and red bars) and insoluble COS (green and yellow bars) pools calculated from the last sample of experimental (black and green bars) or predicted time courses (red and yellow bars). Averages and corresponding standard deviations obtained from models PE18–PE20 are shown. **B**–**E** show time courses of soluble and insoluble COS obtained from experiments (black circles) and predicted by the model (lines). Experiments carried out at a αGlc1-*P*/G2 ratio of 4.0 (**B** and **C**) and 3.0 (**D**, **E**) served as basis. Solid lines indicate representative data predicted by PE18 while dashed lines indicate boundaries of solution spaces obtained from all three models. Of note: Predicted characteristics of COS pools over time in Panel B and C were almost identical for all three models (*R*^2^ = 0.961 ± 0.0002 (**B**), *R*^2^ = 0.965 ± 0.0002 (**C**)). Shown solutions spaces in **D** and **E** were obtained within ranges for *Cc*CdP of 1.0 ± 0.05 mmol/L/min and ± 0.5 mM for both substrates

Using the lowest αGlc1-*P*/G2 ratio of 0.2 (= 10 mM/50 mM), only G3 and G4 were formed (Fig. 2A). Consistent with the iterative nature of the COS synthesis, the G3 was formed slightly faster than the G4. The G2 consumption paralleled the G3 formation. Increase in αGlc1-*P*/G2 ratio to 1.0, 2.0 and 4.0 (Fig. 2, panels B–D in that order) resulted in the formation of G5 and G6 in concentrations that increased dependent on the substrate ratio used. The G2 consumption and the phosphate release also increased, as expected. The time courses of G3 and G4 both passed through a maximum only to decrease later in the reaction. At the highest αGlc1-*P*/G2 ratio of 4.0, the decrease in concentration was observed even for G5. The rate and the extent of the decrease in G3 and G4 were dependent on the αGlc1-*P*/G2 ratio. We analyzed the soluble and insoluble COS pools, both expressed as αGlc1-*P* equivalents incorporated. Comparison of the two pools revealed that the portion of insoluble product increased from effectively zero at αGlc1-*P*/G2 ratios of 0.2 and 1.0 to 16.1 ± 5.7 mol.% and 43 ± 7 mol.% at the higher ratios of 2.0 and 4.0, respectively (Fig. 3A).

### Additional analysis of initial rates

To support the kinetic analysis of time courses with relevant boundary conditions as explained later, we determined a select set of kinetic parameters for the enzyme reaction. In particular, reaction was studied with G2 or G3 when αGlc1-*P* (25 mM) was constant and saturating. Initial rate data and the corresponding fitting results are shown in the Additional file 1: Figure S1. The apparent *K*_M_ for G2 was 6.0 ± 0.2 mM, that for G3 was 21 ± 3 mM. The apparent *k*_cat_ was 15.9 ± 0.6 s^−1^ and 25.5 ± 1.3 s^−1^ for G2 and G3, respectively. These parameters of *Cc*CdP are comparable to reported parameters for other CdP enzymes (e.g., *K*_G2_ 13.2 mM and *k*_cat_G2_ 47.1 s^−1^ for the CdP from *Ruminococcus albus* [25]; *K*_G2_ 0.89 mM and *k*_cat_G2_ 10.1 s^−1^ for the CdP from *C. thermocellum* [34]). In addition, using G2 at a saturating (100 mM) or non-saturating concentration (5 mM), we obtained kinetic parameters for αGlc1-*P*. Based on initial rate data shown in Additional file 1: Figure S2, the *K*_M_ was determined as 0.47 ± 0.24 mM at 100 mM G2 and 0.35 ± 0.15 mM at 5 mM G2.

### Mechanism-based kinetic model of the iterative CdP reaction

An ordered kinetic mechanism with αGlc1-*P* as the first binding substrate was assumed for each reaction of the iterative polymerization of G2 to G12 [25, 27, 28]. The G12 was chosen as the largest COS considering that, as shown previously, the insoluble cellulose from the *Cc*CdP reaction has an average DP of 7–9 depending on the conditions used [6]. G6 is the largest of the soluble COS present in noticeable amount. We concluded, therefore, that the abundance of products of DP larger than 12 would have to be irrelevantly small. The *Cc*CdP used was not active on the insoluble cellulose that precipitates from its reaction with αGlc1-*P* and G2, consistent with previous study stating that the precipitated oligo-cellulose is hardly accessible for further elongation by the CdP [8].

The basic reaction is shown in Eq. 1 for the conversion of G2 to G3 and is analogously used for each further step of the enzymatic polymerization.

1 $$\alpha {\text{Glc1}}-P + {\text{ G2}} \leftrightarrow {\text{G3 }} + {\text{ phosphate}}$$

The rate equation for the enzymatic reaction in the forward direction is shown in Eq. 2.

2 $$V = V_{{{\text{max}}}} \left[ {\alpha {\text{Glc1}} - P\left] {} \right[{\text{G2}}]/(K_{{{\text{iGlc1P}}}} K_{{{\text{G2}}}} + K_{{{\text{Glc1P}}}} \left[ {{\text{G2}}} \right]{\text{ }} + K_{{{\text{G2}}}} } \right[\alpha {\text{Glc1}} - P\left] {{\text{ }} + {\text{ }}} \right[\alpha {\text{Glc1}} - P\left] {} \right[{\text{G2}}])$$

*V* is the initial reaction rate, *V*_max_ is the maximum rate, *K*_iGlc1P_ is the constant for binding of αGlc1-*P* to the free enzyme and *K*_Glc1P_ and *K*_G2_ are the Michaelis constants for αGlc1-*P* and G2, respectively. However, Eq. 2 only would be the correct rate law to describe the conversion of G2 into G3 if there was no further conversion of the G3 into G4, and so on. To account for the iterative nature of the CdP reaction, it is necessary to develop a rate law describing the enzymatic glycosylation from αGlc1-*P* under conditions when multiple acceptor substrates are present at the same time. We considered that an alternative substrate (e.g., G4) affects the kinetics of an enzymatic reaction (e.g., conversion of G2 to G3) in the same way as a competitive inhibitor would affect it, except that the relevant constant describing the inhibition is not an inhibitor binding constant (*K*_i_), but effectively the Michaelis constant (e.g., *K*_G4_) for the enzymatic reaction of the alternative substrate. We therefore expanded Eq. 2, by including the proper inhibition terms for the competition from all other COS that could be glycosylated from αGlc1-*P.* In Eq. 3, ^eff^*K*_G2_ is an effective Michaelis constant that includes the effect from all alternative substrates present in the reaction. For the sake of simplicity, we show in Eq. 3 only the terms for the mainly formed COS. For each reaction (G2–G11), the effective Michaelis constant is defined analogously.

3 $$^{{{\text{eff}}}} K_{{{\text{G2}}}} = K_{{{\text{G2}}}} \left( {{\text{1 }} + {\text{ G3}}/K_{{{\text{G3}}}} + {\text{ G4}}/K_{{{\text{G4}}}} + {\text{ G5}}/K_{{{\text{G5}}}} + {\text{ G6}}/K_{{{\text{G6}}}} \ldots + {\text{ G11}}/K_{{{\text{G11}}}} } \right)$$

Each COS substrate involves its own reaction rate for glycosylation from αGlc1-*P* (*V*_G2_, *V*_G3_, *V*_G4_ …) and the rate of αGlc1-*P* consumption (*V*_Glc1P_) is the sum of the individual COS rates. The rate of phosphate release (*V*_Pi_) equals -*V*_Glc1P_. From the rate equations for the glycosylation of each COS, a set of coupled ordinary differential equations is established based on mass balance.

### Effect of the reaction equilibrium

To describe the full time-course of the enzymatic conversion in solution, it was necessary to consider the chemical equilibrium for the reversible glycosylation reactions [35, 36]. The equilibrium constant (*K*_eq_) for the reaction in Eq. 1 is *K*_eq_ = ([phosphate]^eq^ × [G3]^eq^)/([G2]^eq^ × [αGlc1-*P*]^eq^), with reactant concentrations being at equilibrium, and was defined analogously for all other reactions. Using the online tool eQuilibrator [37] which computes equilibria based on chemical group contribution, we obtained *K*_eq_ estimates for reactions of G2, G3, G4 and G5 in a similar range (2.63–3.58). However, the *K*_eq_ estimates were afflicted with errors of up to 570%, making the calculated numbers inappropriate for direct use. For further analysis including data fitting, we set a value of 3.6 as an upper boundary for the *K*_eq_.

For modeling of the reaction time courses, we chose a simple mass action term to describe the effect of the reverse reaction and so the approach to chemical equilibrium. Equation (4) was used where ^net^*V*_G2_ is the net rate and *V*_G2_ the initial rate of G2 consumption, and Γ is the mass action ratio, expressed for the reaction in Eq. 1 as Γ = ([phosphate]^t^ × [G3]^t^)/([G2]^t^ × [αGlc1-*P*]^t^) and for other reactions in the same way. Reactant concentrations in Γ refer to a certain time during conversion.

4 $$^{{{\text{net}}}} V_{{{\text{G2}}}} = V_{{{\text{G2}}}} ({\text{1 }} - {\Gamma}/K_{{{\text{eq}}}} ).$$

Combining Eqs. 2, 3 and 4, we obtained a full rate equation for each step of the iterative glycosylation. The coupled set of rate equations under the constraint of mass balance describes the polymerization process as a whole and can be used to analyze time courses in Fig. 2 that do not involve product precipitation.

### Constraints in kinetic analysis

A complete summary of the constraints used is given in Table 1. Equations 5 and 6 provide relationships between kinetic parameters (*K*_iGlc1P_, *K*_Glc1P_, *K*_G2_, *K*_G3_) and apparent *K*_M_ values obtained from initial rate experiments described above.

5 $$K_{{\text{M}}} \left( {\alpha {\text{Glc1}} - P} \right){\text{ }} = {\text{ }}\left( {K_{{{\text{Glc1P}}}} + K_{{{\text{iGlc1P}}}} K_{{{\text{G2}}}} /\left[ {{\text{G2}}} \right]} \right)/\left( {K_{{{\text{G2}}}} /\left[ {{\text{G2}}} \right]{\text{ }} + {\text{ 1}}} \right)$$

6 $$K_{{\text{M}}} \left( {{\text{G2}}} \right){\text{ }} = K_{{{\text{G2}}}} \left( {K_{{{\text{iGlc1P}}}} /\left[ {\alpha {\text{Glc1}} - P} \right]{\text{ }} + {\text{ 1}}} \right)/\left( {K_{{{\text{Glc1P}}}} /\left[ {\alpha {\text{Glc1}} - P} \right]{\text{ }} + {\text{ 1}}} \right)$$

**Table 1:** Boundaries applied in parameter estimation analyses

Parameter	LB	UB
CdP reaction model		
*K*_iGlc1P_, [mM]	0.17	0.32
*K*_Glc1P_, [mM]	0.23	0.72
*K*_G2_, [mM]	5.80	6.25
*K*_G3_, [mM]	18.0	24.5
*K*_G4_ (= *K*_G5_—*K*_G11_), [mM]	1.0	100.0
*K*_eq_	0.50	3.60
*k*_2_, [−]	1.60	2.00
*k*_3_, [−]	1.00	5.00
*k*_4_, [−]	1.00	5.00
*k*_5_ (= *k*_6_–*k*_10_), [−]	1.00	5.00
Precipitation		
[G5]^SL^, [mM]	14.35	25.64
[G6]^SL^, [mM]	2.68	4.79
[G7]^SL^, [mM]	0.501	0.896
[G8]^SL^, [mM]	0.0937	0.1674
[G9]^SL^, [mM]	0.0175	0.0313
[G10]^SL^, [mM]	0.00327	0.00585
[G11]^SL^, [mM]	0.000612	0.001093
[G12]^SL^, [mM]	0.000114	0.000204
Experiment		
*V*_max_, [mM/min]		
All	0.95	1.05
[G2]^t=0^, [mM]		
αGlc1-*P*/G2: 0.2, 1.0	49.5	50.5
αGlc1-*P*/G2: 2.0, 4.0	24.5	25.5
[αGlc1-*P*]^t=0^, [mM]		
αGlc1-*P*/G2: 0.2	9.5	10.5
αGlc1-*P*/G2: 1.0, 2.0	49.5	50.5
αGlc1-*P*/G2: 4.0	99.5	100.5

*LB* lower boundaries, *UB* upper boundaries

Equation 6 was applied analogously to G3. A further constraint, dictated by the ordered mechanism of CdP [25–27], was that *K*_iGlc1P_ and *V*_max_/*K*_Glc1P_ (the catalytic efficiency for αGlc1-*P*) are the same for all COS substrates (G2–G11). The constraint on *V*_max_/*K*_Glc1P_ was implemented by way of scaling factors (*k*_1_–*k*_5_) that normalized the *V*_max_ and *K*_Glc1P_ for each COS substrate (G3–G6; *k*_2_–*k*_5_) on the *V*_max_ and *K*_Glc1P_ for G2 (*k*_1_ = 1.0). For COS substrates larger than G6, the scaling factor was assumed as *k*_5_. Lastly, since CdP exhibits only 3 sugar-binding subsites (+ 1–+ 3) to position the COS substrate for glycosylation from αGlc1-*P* [27], we assumed that the Michaelis constant for COS of DP ≥ 5 was the same as *K*_G4_ (Table 1).

Preliminary fitting analysis (data not shown) revealed that the kinetic model developed to this point was able to describe well the experimental time courses of G3 and G4 for reaction at the lowest substrate ratio (αGlc1-*P*/G2 = 0.2) that effectively prohibits the insoluble product formation (Fig. 2A). We thus were encouraged to consider kinetic modeling of the COS precipitation.

### Hybrid model including DP-dependent precipitation of the COS

COS solubility dependent on the DP was taken from literature (G3–G5) and extrapolated from the available data to higher DPs (G5–G12) [5]. As shown in Additional file 1: Figure S3, the solubility followed a linear trend (*R*^2^ = 0.93) to decrease as the DP increases. For modeling, the solubility of G5–G12 was allowed to vary between boundaries of 0.72 and 1.28 times the value obtained by extrapolation (Table 1). Precipitation of the COS was modeled as a first-order reaction, shown in Eq. 7 for G5 and used for other COS of DP ≥ 6 analogously.

7 $$^{{{\text{prec}}}} V_{{{\text{G5}}}} = - k_{{{\text{G5}}}} {\text{[G5]}}$$

The precipitation was modeled empirically as a kinetic process controlled by the precipitation rate constant. The rate constant varied between zero and its maximum value (^max^*k*_G5_) dependent on the COS concentration in relation to the COS solubility limit, as shown in Eq. 8 for the example of *k*_G5_

8 $${k_{{\text{G}}5}} = {{^{\max}{k_{{\text{G}}5}}} \mathord{\left/ {\vphantom {{^{\max }{k_{{\text{G}}5}}} {\left[ {1 + \exp \left( {{{\left( {{{\left[ {{\text{G}}5} \right]}^{{\text{SL}}}} - {{\left[ {{\text{G}}5} \right]}^t}} \right)} \mathord{\left/ {\vphantom {{\left( {{{\left[ {{\text{G}}5} \right]}^{{\text{SL}}}} - {{\left[ {{\text{G}}5} \right]}^t}} \right)} a}} \right. \kern-\nulldelimiterspace} a}} \right)} \right]}}} \right. \kern-\nulldelimiterspace} {\left[ {1 + \exp \left( {{{\left( {{{\left[ {{\text{G}}5} \right]}^{{\text{SL}}}} - {{\left[ {{\text{G}}5} \right]}^t}} \right)} \mathord{\left/ {\vphantom {{\left( {{{\left[ {{\text{G}}5} \right]}^{{\text{SL}}}} - {{\left[ {{\text{G}}5} \right]}^t}} \right)} a}} \right. \kern-\nulldelimiterspace} a}} \right)} \right]}}.$$

In Eq. 8, [G5]^SL^ is the G5 solubility limit and [G5]^t^ is the soluble G5 concentration at a certain time. The parameter *a* relative to the solubility limit determines the steepness of the increase from zero to ^max^*k*_G5_. We set the *a* (G5) to have a value of 10^–4^, to ensure a sharp increase in the precipitation rate once 99.99% of the solubility limit was reached. *k*_G5_ is 50% of the ^max^*k*_G5_ at the solubility limit and approaches 99.995% of ^max^*k*_G5_ at 100.005% of the solubility limit (Additional file 1: Figure S4). To ensure the same precipitation dynamics for each COS, the parameters *a* and ^max^*k*_G_ had to be adjusted based on relative solubility limit. Therefore, the parameter *a* (Gn) was scaled from *a* (G5) with the ratio [Gn]^SL^/[G5]^SL^ and ^max^*k*_G_ (Gn) was scaled from ^max^*k*_G5_ with the inverse ratio [G5]^SL^/[Gn]^SL^. The ^max^*k*_G5_ was assumed to be fast and its value set to 10^6^ 1/min.

The mechanistic model of *Cc*CdP-catalyzed polymerization of COS chains in solution was thus expanded into a hybrid model that included the empirical description of COS precipitation.

### Data fitting and parameter estimation

The mechanistic model of the CdP reaction involved 9 unique kinetic parameters and 1 thermodynamic parameter (*K*_eq_) for estimation. Table 1 shows these parameters together with the associated upper and lower boundaries applied in data fitting. Initial conditions for the substrate concentrations and the *Cc*CdP activity (*V*_max_ for G2) were allowed to vary by experimental error estimated to be ± 0.5 mM and ± 0.05 mM/min, respectively (Table 1). Data fitting was done using simultaneously all 22 time courses from Fig. 2 that represent four different synthesis experiments. Parameter estimation was done in 3 independent fittings (models PE18–PE20) and consistent results were obtained for all parameters (Table 2). The fitted time courses are shown together with the experimental data in Fig. 2. To evaluate the overall quality of the models we created and analyzed the correlation plots (Additional file 1: Figure S5) and calculated bias and accuracy factors (Additional file 1: Table S1) [38]. The overall fit quality of model PE18–PE20 was hardly distinguishable with respect to correlation parameters (*R*^2^ (= 0.991); slope value (= 0.991) and root mean square error (RMSE) (= 1.323–1.326)), bias factor (= 1.01) and accuracy factor (= 1.14). Obtained values of model quality parameters imply that the three models are comparable; and they represent experimental data with low bias (1.0%) and at high accuracy (predicted data are only by a factor of 1.14 different from the observed value). The correlation coefficient *R*^2^ for the fit of the respective time course (G2–G6, phosphate) in each experiment is summarized for each model in Table 3. Again, the fit quality of model PE18–PE20 was almost identical and *R*^2^ differed by only ≤ 0.51%. With the exception of G6 in experiments carried out at an initial αGlc1-*P*/G2 ratio of 2.0 (*R*^2^ = 0.6833 ± 0.0035) and 4.0 (*R*^2^ = 0.7556 ± 0.0014), the *R*^2^ exceeded a value of 0.857 and generally was 0.90 or higher (Table 3). This indicated the model fit to have been very good and to cover the dynamics of each individual COS appropriately. Therefore, the models predicted equally well the soluble and insoluble COS pools accumulated in total (Fig. 3A) and over time, as can be seen in panels B and C of Fig. 3. The predicted composition of the insoluble product (G6: 44%, G7: 30%, G8: 22%, G9: 4%, G10: 1%; by mol), with an average DP of 7.0 (1145.7 Da), was in good accordance with experimental findings.

**Table 2:** Summary of results obtained from parameter estimation analyses

Parameter	PE18	PE19	PE20
	Value	Value	Value
CdP reaction			
*K*_iGlc1P_, [mM]	0.32	0.32	0.32
*K*_Glc1P_, [mM]	0.72	0.72	0.72
*K*_G2_, [mM]	6.22	6.23	6.14
*K*_G3_, [mM]	24.5	24.5	24.5
*K*_G4_ (= *K*_G5_–*K*_G11_), [mM]	98.6	99.1	58.5
*K*_eq_	1.03	1.03	1.04
*k*_2_, [−]	1.94	1.94	1.98
*k*_3_, [−]	4.57	4.59	2.80
*k*_4_, [−]	4.31	4.33	2.66
*k*_5_ (= *k*_6_–*k*_10_), [−]	1.56	1.56	1.00
Precipitation			
[G5]^SL^, [mM]	14.73	14.35	14.59
[G6]^SL^, [mM]	2.68	2.68	2.68
[G7]^SL^, [mM]	0.896	0.896	0.896
[G8]^SL^, [mM]	0.1620	0.1674	0.1672
[G9]^SL^, [mM]	0.0308	0.0313	0.0313
[G10]^SL^, [mM]	0.00331	0.00333	0.00585
[G11]^SL^, [mM]	0.001084	0.001087	0.000706
[G12]^SL^, [mM]	0.000114	0.000196	0.000114

**Table 3:** Collection of *R*^2^ values obtained from fitting time courses generated for experiments A–D with models PE18, PE19 and PE20

Model	Experiment αGlc1-*P*/G2	Cellobiose	G3	G4	G5	G6	Phosphate
PE18	0.2	0.904	0.908	0.869			0.966
PE19		0.904	0.908	0.868			0.966
PE20		0.904	0.908	0.866			0.966
PE18	1.0	0.993	0.920	0.965	0.919	0.942	0.973
PE19		0.994	0.919	0.965	0.919	0.942	0.973
PE20		0.992	0.921	0.966	0.921	0.941	0.972
PE18	2.0	0.905	0.937	0.987	0.881	0.686	0.976
PE19		0.905	0.937	0.987	0.882	0.685	0.976
PE20		0.905	0.937	0.988	0.886	0.679	0.977
PE18	4.0	0.967	0.960	0.990	0.969	0.756	0.993
PE19		0.967	0.961	0.990	0.969	0.754	0.993
PE20		0.967	0.960	0.990	0.970	0.757	0.993

Parameter estimates are summarized in Table 2. The correlation matrix associated with these estimates is shown in Additional file 1: Table S2. The maximum reaction rate with G3 was about twofold that with G2. It was further increased about 2.7-fold and 4.6-fold when G4 and G5 was the substrate, respectively. For COS with a DP ≥ 6, the *V*_max_ was assumed to be constant. The fitting results showed this *V*_max_ to be enhanced not at all, or just slightly (~ 1.55-fold), as compared to the *V*_max_ for G3. The *K*_iGlc1P_ (αGlc1-*P* binding constant) and the *K*_Glc1P_ (αGlc1-*P* Michaelis constant) were estimated at the respective upper boundaries of the fit. The *K*_G2_ was well determined whereas the *K*_G3_ and the *K*_G4_ (= *K*_G5_–*K*_G11_) were all at their respective upper boundary, with *K*_G4_ in model PE20 as the sole exception (Table 2). The reason for parameter estimates at upper boundary was identified from Additional file 1: Table S2 which shows strong statistical correlation between the estimate of *K*_G2_ and the estimates of *K*_G3_ and *K*_G4_ as well as the estimated scaling factors *k*_1_–*k*_5_. These results emphasize the importance of additional data from complementary initial rate studies (Additional file 1: Figures S1 and S2) to provide unique constraints for the time-course fitting. It is interesting that the *K*_G2_ was considerably smaller than both the *K*_G3_ and the *K*_G4_. Therefore, occupancy of subsite + 3 (G3) and subsites + 3/ + 4 (G4) did not lead to stronger apparent binding of the acceptor oligosaccharide compared to G2. The *K*_eq_ was well determined from the model fit. Its estimated value was 1.037 ± 0.004.

### Model verification by simulation

**Fig. 4:**
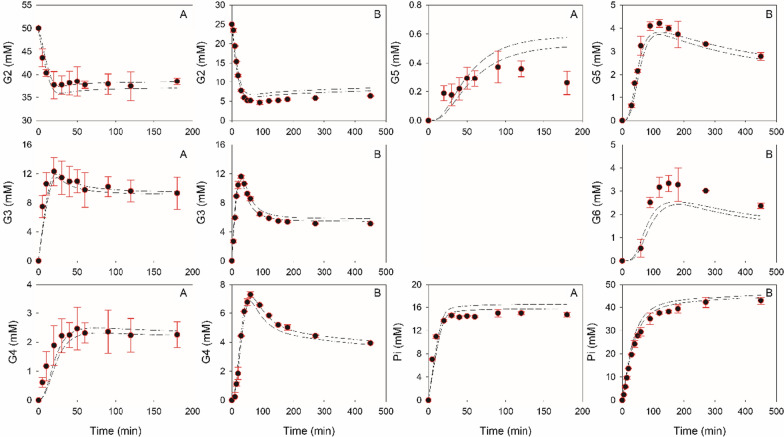
Comparison of time courses obtained from experiments with corresponding solution spaces predicted by models PE18–PE20. **A**, **B** refer to experiments carried out at a αGlc1-*P* to G2 ratio of 0.4 (20 mM αGlc1-*P* and 50 mM G2) and 3.0 (75 mM αGlc1-*P* and 25 mM G2). Boundary conditions to estimate the solution space were: 0.95–1.05 mM/min *Cc*CdP and ± 0.5 mM initial substrate concentration. Boundaries of solution spaces are shown as dashed lines

To verify the hybrid model of the *Cc*CdP reaction as obtained by fitting (Table 1, Fig. 2), we performed new synthesis experiments and used all three models to simulate the time courses of G2 consumption and product formation. The experimental conditions involved new αGlc1-*P*/G2 substrate ratios that lead to primarily soluble products (ratio: 0.4) and involve product precipitation in large amount (ratio: 3.0). Upper and lower bounds obtained for the simulated time courses are shown superimposed on the experimental data in Fig. 4. There was excellent reproduction of the experiments by the hybrid model, with exceptions noted only for the time course of G5 and G6 at a αGlc1-*P*/G2 ratio of, respectively, 0.4 and 3.0. Figure 3D, E compares the simulated solution space for the time-dependent formation of the pools of soluble (panel D) and insoluble COS (panel E) with the corresponding experimental data obtained from reaction at αGlc1-*P*/G2 ratio of 3.0. The soluble COS were predicted well. For the insoluble COS, the overall trend was captured correctly. It is worth noting that the simulation solution space resulted solely from the allowed variation in the initial conditions for substrate concentrations and enzyme loading. It was not due to difference in the models PE18–PE20.

### Model-based analysis and optimization

**Fig. 5:**
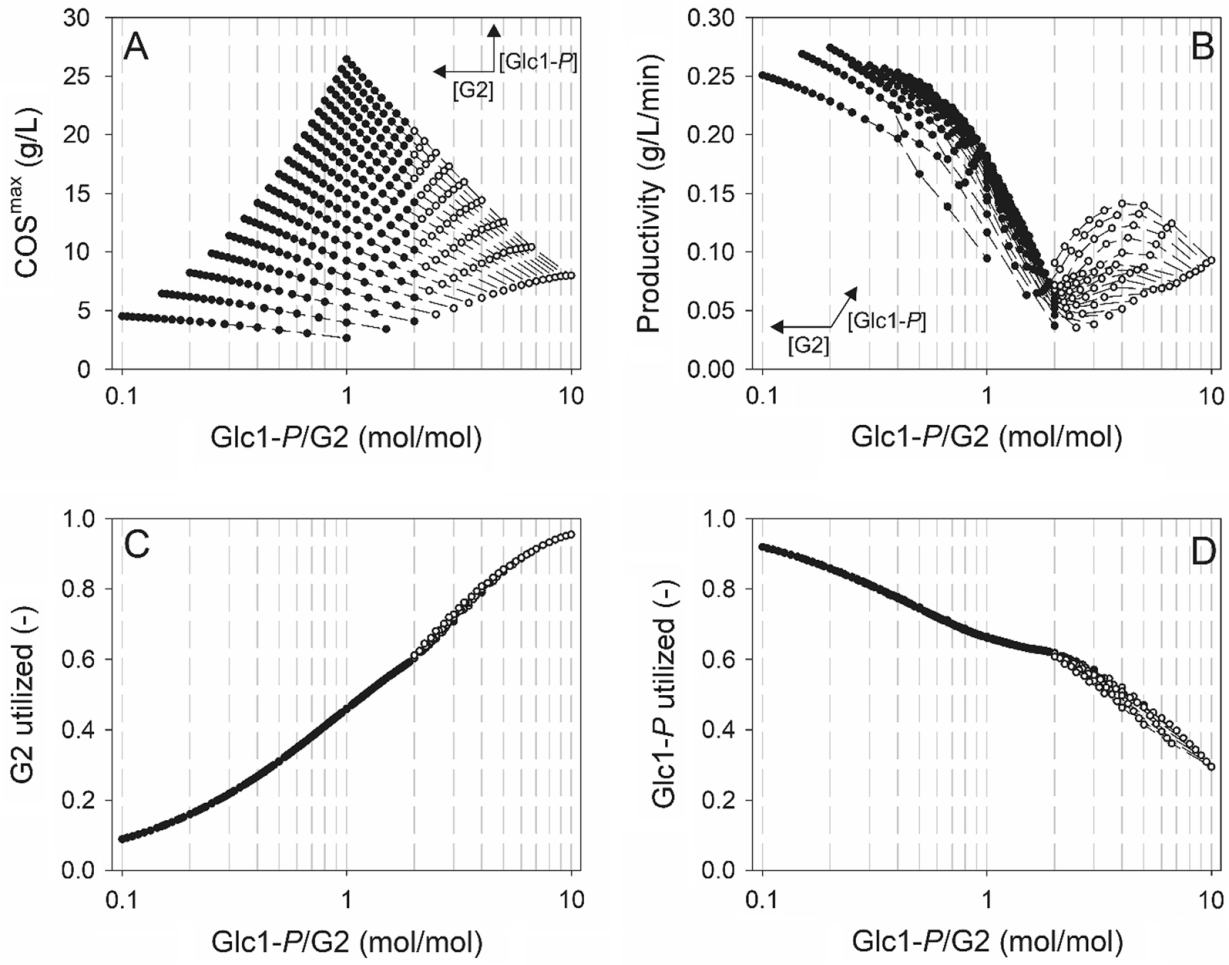
Maximal soluble COS titers (COS^max^, **A**), productivities (**B**) and corresponding substrate utilization efficiency (Panel C and D) shown in relation to the αGlc1-*P*/G2 ratio. Full circles and empty circles indicate soluble COS^max^ fractions without and with co-production of insoluble COS, respectively. Dashed lines correspond to data obtained at identical initial αGlc1-*P* concentrations. Variation of αGlc1-*P* and G2 concentrations within the substrate range screened (10–100 mM, with 5 mM intervals for both substrates) are indicated in **A**, **B**

The hybrid model PE18 with parameters from Table 2 was used for computational window-of-operation analysis to facilitate optimization of the enzymatic synthesis of soluble COS. As models PE18–PE20 had yielded identical results in simulations (substrate consumption, formation of soluble and insoluble COS), window-of-operation analysis with only one of the three models was considered to be sufficient. In the example chosen, the concentrations of αGlc1-*P* and G2 were varied each at 5-mM intervals between 10 and 100 mM. The enzyme activity was 1 U/mL and the reaction time was scanned at 2-min intervals up to 500 min. The processing objective was the maximum soluble COS concentration ([COS]^max^; g/L) for each condition used. Results are shown in a condensed form in Fig. 5. They are presented fully in the Excel file in the Additional file 2. We show in Fig. 5A that the [COS]^max^ increased with increasing ratio of αGlc1-*P*/G2; and that it also increased with increasing αGlc1-*P* concentration at constant αGlc1-*P*/G2 ratio. The calculated [COS]^max^ was highest (26.435 g/L) at the maximum loading of both substrates (100 mM). While the evidence on [COS]^max^ might seem as expected, analysis of additional parameters of reaction output revealed complex interplay of factors that only the modeling could disentangle for clear insight. The productivity ([COS]/∆t) associated with the formation of [COS]^max^ was 1.5-fold lower (0.181 g/L/min) compared to the maximal productivity (0.274 g/L/min) found within the searched concentration range. As shown in Fig. 5B, the productivity exhibited complex dependence on the αGlc1-*P*/G2 ratio. Interestingly, the productivity showed a pronounced minimum at αGlc1-*P*/G2 of ~ 2.0, only to increase at lower and higher substrate ratios. The productivity increases at αGlc1-*P*/G2 ratios greater than 2.0 involved an additional promoting effect of high αGlc1-*P* concentration (Fig. 5B). Figure 5 furthermore shows that the G2 conversion efficiency (panel C) increased with increasing αGlc1-*P*/G2 ratio whereas the αGlc1-*P* conversion efficiency (panel D) exhibited the opposite trend dependence. Additional observation of interest concerned the release of insoluble COS. Figure 5A shows that the [COS]^max^ were accompanied by insoluble COS when the αGlc1-*P*/G2 ratio exceeded a value of ~ 2.0. The plots of productivity (Fig. 5B) and substrate conversion efficiency (Fig. 5C/D) also emphasize the transition to formation of insoluble product dependent on the αGlc1-*P*/G2 ratio. The product released at [COS]^max^ was composed of G3 (56.3%), G4 (32.7%), G5 (9.2%) and G6 (1.7%) by weight. At maximum productivity, the COS product was composed of G3 (90.3%), G4 (9.3%) and G5 (0.4%) by weight. In summary, the window-of-operation analysis here exemplified can quickly identify reaction conditions (substrate concentration, substrate ratio, reaction time) aligned to the immediate task of the biocatalytic synthesis (e.g., avoidance of insoluble product). The maximum [COS] for each condition can be combined with the usage efficiency of αGlc1-*P* and G2 to identify point(s) of practical operation.

**Fig. 6:**
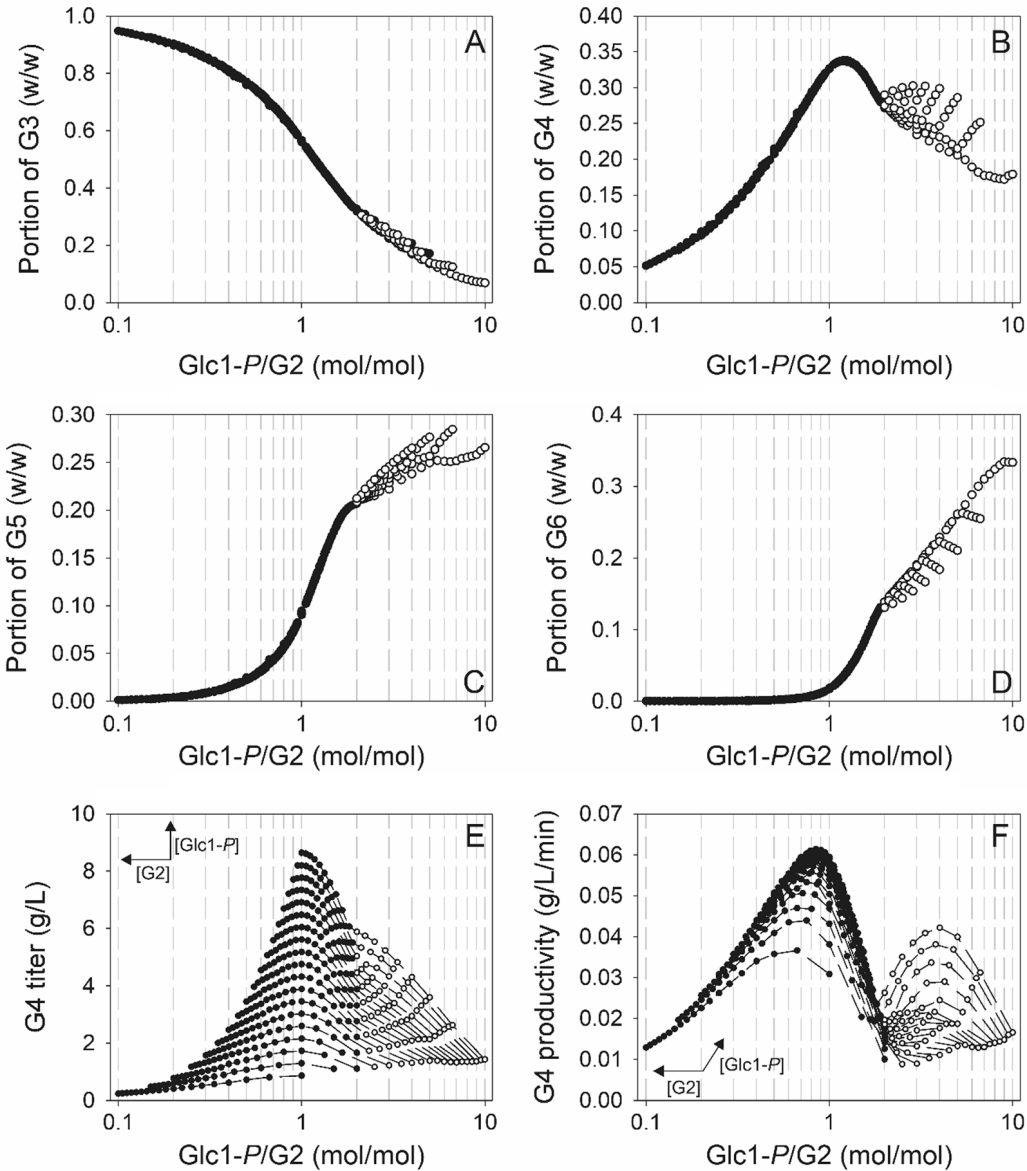
Relative enrichment of G3–G6 in COS^max^ fractions (**A**–**D**) and window-of-operation to produce G4 enriched COS^max^ fractions (**E**, **F**) depending on the αGlc1-*P*/G2 ratio. Full circles and empty circles indicate soluble COS^max^ fraction without and with co-production of insoluble COS, respectively. **E**, **F** Dashed lines correspond to data obtained at identical initial αGlc1-*P* concentrations. Variation of αGlc1-*P* and G2 concentrations within the substrate range screened (10–100 mM, with 5 mM intervals for both substrates) is indicated by arrows

We then moved on to analyze the COS composition. Figure 6 shows for each oligosaccharide of DP3 to DP6 on the basis of mass, how the relative portion in total COS changes dependent on the αGlc1-*P*/G2 ratio. Whereas the G3 portion decreased continuously with increasing αGlc1-*P*/G2 (Fig. 6A), the G4 portion passed through a distinct maximum of ~ 0.34 at a αGlc1-*P*/G2 ratio of 1.2 (Fig. 6B). The G5 portion was not significant up to a αGlc1-*P*/G2 ratio of ~ 0.2 (Fig. 6C). It increased sharply at higher αGlc1-*P*/G2 ratios, reaching a portion of ~ 0.2 at αGlc1-*P*/G2 of 1.9–2.0. Due to the onset of insoluble product formation at αGlc1-*P*/G2 greater than 2.0, increase in the relative portion of G5 in total COS product was attenuated. The G6 formation started to be significant at a αGlc1-*P*/G2 ratio of ~ 0.5. The portion G6 increased to value of ~ 0.13 before insoluble COS are co-produced (Fig. 6D).

In a next step, we were interested in a more detailed analysis of the G4 and show the calculated concentration (Fig. 6E) and productivity (Fig. 6F) of G4 dependent on the αGlc1-*P*/G2 ratio. The αGlc1-*P*/G2 ratio giving maximum [G4] was ~ 1.0, that giving maximum productivity was ~ 0.84. Comparable to the trend for [COS]^max^ (Fig. 5B), the productivity of G4 displayed a minimum at a αGlc1-*P*/G2 ratio of 2.0. From these results, conditions can be selected that tune the concentration of G4 product or the relative portion of it in the total COS released. Importantly, predicted relationships identified through window-of-operation analysis would not have been accessible without the model.

## Conclusions

In a novel approach of kinetic modeling, we herein developed a hybrid kinetic model for the iterative β-1,4-glycosylation of G2 from αGlc1-*P* catalyzed by *Cc*CdP. The hybrid model combined a detailed mechanism-based description of kinetic and thermodynamic characteristics of the enzymatic reaction in multiple steps with an empirical description of the spontaneous (uncatalyzed) self-assembly of COS into insoluble material. The hybrid model involved 9 parameters that were estimated from reaction time-course fits performed with well-defined constraints derived from accompanying initial rates studies. Simulations showed the hybrid model to predict the time courses of further experiments, not previously used in fitting, with excellent quality. Model simulations were also used in a window-of-operation analysis to study and optimize the biocatalytic synthesis of COS. Key process parameters (i.e., yield, COS concentration, composition of soluble COS) were predicted in dependence of the main process variables (substrate and enzyme concentrations, αGlc1-*P*/G2 ratio, reaction time). It was shown that a αGlc1-*P*/G2 ratio of ~ 2.0 marked the transition in the reaction to insoluble product formation. This ratio was also the point of lowest productivity of the soluble COS.

The mechanistic part of the hybrid model can be relevant to other enzymatic reactions used to synthesize product oligomers via iterative polymerization. Various phosphorylases (e.g., α-glucan phosphorylase [19], laminarin phosphorylase [16, 17], β-glucan phosphorylase [18]) catalyze the polymerization of a primer substrate from αGlc1-*P*. Sugar nucleotide-dependent glycosyltransferases [22, 23] catalyze the polymerization of neutral and acidic oligosaccharides. These oligosaccharides have considerable interest for technological application in different industrial sectors, including food and feed, cosmetics and health-care. The current study provides an important resource of methodology for the kinetic analysis of the enzymatic polymerization; and it shows use of the model for reaction analysis and optimization.

## Methods

Unless stated, chemicals were of highest purity available from Sigma-Aldrich (Vienna, Austria) or Carl Roth (Karlsruhe, Germany). COS standards of DP 3 to 6 (purity ≥ 95%; cellotetraose purity ≥ 90%) were from Megazyme (Wicklow, Ireland). Cellobiose (purity ≥ 99%) was from Pfeifer & Langen (Köln, Germany).

### Enzyme

*N*-terminally His-tagged *Cc*CdP (GenBank identifier CDZ24361.1) expressed and purified according to reported method [6] was used in all experiments. Enzyme stock solution (~ 20 mg protein/mL) was made in 50 mM MES buffer (pH 7.0). It was stored at − 20 °C for several weeks, and could be thawed and frozen repeatedly, without significant loss of activity (data not shown). Enzyme purity was verified by SDS PAGE and determination of specific activity (13.3 ± 0.4 U/mg purified protein). Activity determination was performed according to a reported method [6]. Briefly, enzymatic reaction containing cellobiose and αGlc1-*P* (each 50 mM) was analyzed at 45 °C in 50 mM MES buffer (pH 7.0). One unit (U) of activity is the enzyme amount releasing 1 μmol phosphate/min under standard assay conditions. Protein was measured with Roti-Quant reagent (Carl Roth, Karlsruhe, Germany) using bovine serum albumin as standard.

### Iterative glycosylation reactions

Reactions were carried out at 45 °C and 300 rpm agitation rate on a ThermoMixer C (Eppendorf, Vienna, Austria). The total volume was 1.0 mL and *Cc*CdP was used at 0.08 mg/mL (1 U/mL) in MES buffer (50 mM, pH 7.0). The reaction time was up to 500 min. The enzyme was fully stable during that time under the conditions used. The concentrations of αGlc1-*P* and G2 were varied as indicated in the “Results and discussion” section. Briefly, the reactions with a αGlc1-*P*/G2 ratio of 0.2 (10 mM/50 mM), 0.4 (20 mM/50 mM) and 1.0 (50 mM/50 mM) were done to produce COS products that were mainly soluble. By contrast, reactions with a αGlc1-*P*/G2 ratio of 2.0 (50 mM/25 mM), 3.0 (75 mM/25 mM), and 4.0 (100 mM/25 mM) were done to also form COS products that were insoluble. Samples (100 µL) were taken at certain times, heated (95 °C, 5 min), centrifuged (21,130 × *g*, 4 °C, 10 min), and the supernatant was analyzed. Each reaction was carried out in duplicates.

### Analytics

For quantification of soluble COS, measurement was done on a Hitachi LaChrom HPLC system (Merck, Darmstadt, Germany) equipped with a Luna 5 µm NH_2_ column (100 Å, 250 × 4.6 mm, Phenomenex, Aschaffenburg, Germany) operated at 40 °C. Acetonitrile–water (70:30, volume ratio) was used as eluent at a flow rate of 1.5 mL/min. Refractive index detection was used. Calibration was done with authentic standards of G2 (2.5–50 mM), G3/G4 (2.5–15 mM), G5 (1.0–7.5 mM) and G6 (1.0–2.5 mM). The phosphate release was measured using colorimetric assay [39].

The measured concentration of substrate and products were assessed for internal consistency based on mass balance. Considering the reactions in which a substantial portion of products ended up insoluble, a so-called soluble mole ratio (mol.%) was defined. This is the ratio of glucosyl units in soluble products to the total glucosyl units transferred from αGlc1-*P* in the overall reaction. The released phosphate equals the αGlc1-*P* converted and the glucosyl residues not accounted for in the soluble products are considered to be present as insoluble products. The experimentally traceable soluble COS are limited to DP ≤ 6. Glucosyl residues not found in G3–G6 are thus taken as insoluble COS. The model-derived data were therefore treated in exactly the same way.

### Initial rate kinetic analysis

This was performed to determine the selected kinetic parameters as constraints for the fitting of reaction time courses. Duplicate reactions (50 mM MES, pH 7.0) used a total volume of 200 µL and an enzyme concentration of 0.014 mg/mL. Incubations were done at 45 °C and 400 rpm agitation rate on a ThermoMixer C (Eppendorf). Acceptor concentrations (cellobiose 1.0–40 mM; cellotriose 5.0–80 mM) were varied at a fixed αGlc1-*P* concentration (25 mM). The αGlc1-*P* concentrations (0.2–20 mM) were varied with fixed cellobiose concentrations of 5 mM and 100 mM. The phosphate release within 5 min was measured and initial rates calculated from the data. The phosphate release was linear with time. Initial rates were plotted against the varied substrate concentration and analyzed with the Michaelis–Menten equation by non-linear regression fitting (GraphPad Prism 9; https://www.graphpad.com/scientific-software/prism/). The apparent Michaelis constant (*K*_M_) and the maximal velocity (*V*_max_) were calculated.

### Kinetic modeling

The freely available software Copasi: Biochemical System Simulator 4.29 (Build 228) was used for data fitting, simulation and window-of-operation analysis [40]. Differential evolution with a population size of 43 was used in parameter estimation. Typically, about 2.8 × 10^6^ function evaluations were performed before the objective value reached a minimum. Averages obtained from duplicate experiments were used in the fitting procedure and also to calculate reactant-specific correlation coefficients *R*^2^.

To evaluate the quality of the kinetic models, correlation plots of predicted versus observed data were generated and analyzed by linear regression and regression parameters *R*^2^, slope value and root mean square error (RMSE) were determined. Furthermore, to address the performance of a model bias and accuracy factors were calculated in accordance with the reported literature [38]. Both factors are 1.0 if predictions from a model perfectly match the experimental data. Analyses were carried out for models PE18–PE20 and the complete experimental data set inclusively variation was considered (*n* = 765).

The Excel solver was used in the analysis (e.g., optimization) of algebraic relationships. The solver was applied to minimize the sum of squared differences between calculated and experimentally determined values. The procedure was used to calculate boundary conditions for *K*_iGlc1P_, *K*_Glc1P_, *K*_G2_, and *K*_G3_ using Eqs. 5 and 6 in combination with experimentally determined *K*_M_ values. The upper and lower boundaries resulted from the standard deviations of *K*_M_. Standard rules were used to account for error propagation throughout the calculations [41].

## Supplementary Information

**Additional file 1.** Initial rate kinetic analyses of enzyme (*Cc*CdP) towards acceptors (cellobiose or cellotriose) and donor (αGlc1-*P*): **Figure S1 and S2**; Semi logarithmic relationship between solubilities of COS and their respective number of glucose molecules: **Figure S3**; COS precipitation dynamics: **Figure S4**; Comparison of experimental data from time course analyses with data obtained from model predictions: **Figure** **S5**. Results obtained from evaluating model quality: **Table S1**; Correlation matrix of fitted parameters: **Table S2.**

**Additional file 2.** Summary of window-of-operation analysis of enzymatic synthesis of soluble COS.

## Data Availability

The datasets generated and/or analyzed during the current study are available from the https://doi.org/10.5281/zenodo.4555807.

## Abbreviations

CdP: Cellodextrin phosphorylase (EC 2.4.1.49)
*Cc*CdP: CdP from *Clostridium cellulosi*
COS: Cello-oligosaccharide(s)
DP: Degree of polymerization
αGlc1-*P*: α-d-Glucose 1-phosphate
LB: Lower boundaries
RMSE: Root mean square error
UB: Upper boundaries

## References

[1] Kitaoka M, Hayashi K (2002). Carbohydrate-processing phosphorolytic enzymes. Trends Glycosci Glycotechnol.

[2] Puchart V (2015). Glycoside phosphorylases: structure, catalytic properties and biotechnological potential. Biotechnol Adv.

[3] Billès E, Coma V, Peruch F, Grelier S (2017). Water-soluble cellulose oligomer production by chemical and enzymatic synthesis: a mini-review. Polym Int.

[4] Shrotri A, Lambert LK, Tanksale A, Beltramini J (2013). Mechanical depolymerisation of acidulated cellulose: understanding the solubility of high molecular weight oligomers. Green Chem.

[5] Taylor JB (1957). The water solubilities and heats of solution of short chain cellulosic oligosaccharides. Trans Faraday Soc.

[6] Zhong C, Luley-Goedl C, Nidetzky B (2019). Product solubility control in cellooligosaccharide production by coupled cellobiose and cellodextrin phosphorylase. Biotechnol Bioeng.

[7] Hata Y, Sawada T, Serizawa T (2018). Macromolecular crowding for materials-directed controlled self-assembly. J Mater Chem B.

[8] Petrovic DM, Kok I, Woortman AJ, Ciric J, Loos K (2015). Characterization of oligocellulose synthesized by reverse phosphorolysis using different cellodextrin phosphorylases. Anal Chem.

[9] Kitaoka M (2015). Diversity of phosphorylases in glycoside hydrolase families. Appl Microbiol Biotechnol.

[10] Lynd LR, Weimer PJ, van Zyl WH, Pretorius IS (2002). Microbial cellulose utilization: fundamentals and biotechnology. Microbiol Mol Biol Rev.

[11] Hiraishi M, Igarashi K, Kimura S, Wada M, Kitaoka M, Samejima M (2009). Synthesis of highly ordered cellulose II in vitro using cellodextrin phosphorylase. Carbohydr Res.

[12] Pylkkänen R, Mohammadi P, Arola S, de Ruijter JC, Sunagawa N, Igarashi K, Penttilä M (2020). In vitro synthesis and self-assembly of cellulose II nanofibrils catalyzed by the reverse reaction of *Clostridium thermocellum* cellodextrin phosphorylase. Biomacromol.

[13] Zhong C, Ukowitz C, Domig K, Nidetzky B (2020). Short-chain cello-oligosaccharides: intensification and scale-up of their enzymatic production and selective growth promotion among probiotic bacteria. J Agric Food Chem.

[14] Karnaouri A, Matsakas L, Krikigianni E, Rova U, Christakopoulos P (2019). Valorization of waste forest biomass toward the production of cello-oligosaccharides with potential prebiotic activity by utilizing customized enzyme cocktails. Biotechnol Biofuels.

[15] Serizawa T, Fukaya Y, Sawada T (2018). Nanoribbon network formation of enzymatically synthesized cellulose oligomers through dispersion stabilization of precursor particles. Polym J.

[16] Ogawa Y, Noda K, Kimura S, Kitaoka M, Wada M (2014). Facile preparation of highly crystalline lamellae of (1→3)-β-d-glucan using an extract of *Euglena gracilis*. Int J Biol Macromol.

[17] Kuhaudomlarp S, Patron NJ, Henrissat B, Rejzek M, Saalbach G, Field RA (2018). Identification of *Euglena gracilis* β-1,3-glucan phosphorylase and establishment of a new glycoside hydrolase (GH) family GH149. J Biol Chem.

[18] Abe K, Nakajima M, Kitaoka M, Toyoizumi H, Takahashi Y, Sugimoto N, Nakai H, Taguchi H (2015). Large-scale preparation of 1,2-β-glucan using 1,2-β-oligoglucan phosphorylase. J Appl Glycosci.

[19] Kadokawa J-I (2018). α-Glucan phosphorylase-catalyzed enzymatic reactions using analog substrates to synthesize non-natural oligo- and polysaccharides. Catalysts.

[20] Liu J, Linhardt RJ (2014). Chemoenzymatic synthesis of heparan sulfate and heparin. Nat Prod Rep.

[21] Plou FJ, Segura AGd, Ballesteros A. Application of glycosidases and transglycosidases in the synthesis of oligosaccharides. In: Polaina J, MacCabe AP, editors. Industrial enzymes: structure, function and applications. Dordrecht: Springer Netherlands; 2007, pp. 141–157.

[22] Weijers CA, Franssen MC, Visser GM (2008). Glycosyltransferase-catalyzed synthesis of bioactive oligosaccharides. Biotechnol Adv.

[23] Nidetzky B, Gutmann A, Zhong C (2018). Leloir glycosyltransferases as biocatalysts for chemical production. ACS Catal.

[24] Nidetzky B, Zhong C. Phosphorylase-catalyzed bottom-up synthesis of short-chain soluble cello-oligosaccharides and property-tunable cellulosic materials. Biotechnol Adv. 2020;107633. 10.1016/j.biotechadv.2020.107633 32966861

[25] Sawano T, Saburi W, Hamura K, Matsui H, Mori H (2013). Characterization of *Ruminococcus albus* cellodextrin phosphorylase and identification of a key phenylalanine residue for acceptor specificity and affinity to the phosphate group. FEBS J.

[26] Kitaoka M, Taniguchi H, Hayashi K (2002). Characterization of cellobiose phosphorylase and cellodextrin phosphorylase. J Appl Glycosci.

[27] O'Neill EC, Pergolizzi G, Stevenson CEM, Lawson DM, Nepogodiev SA, Field RA (2017). Cellodextrin phosphorylase from *Ruminiclostridium thermocellum*: X-ray crystal structure and substrate specificity analysis. Carbohydr Res.

[28] Hidaka M, Honda Y, Kitaoka M, Nirasawa S, Hayashi K, Wakagi T, Shoun H, Fushinobu S (2005). Reaction mechanism and substrate recognition of GH-94 phosphorolytic enzymes. J Appl Glycosci.

[29] Shintate K, Kitaoka M, Kim Y-K, Hayashi K (2003). Enzymatic synthesis of a library of β-(1→4) hetero-d-glucose and d-xylose-based oligosaccharides employing cellodextrin phosphorylase. Carbohydr Res.

[30] Nakai H, Hachem MA, Petersen BO, Westphal Y, Mannerstedt K, Baumann MJ, Dilokpimol A, Schols HA, Duus JO, Svensson B (2010). Efficient chemoenzymatic oligosaccharide synthesis by reverse phosphorolysis using cellobiose phosphorylase and cellodextrin phosphorylase from *Clostridium thermocellum*. Biochimie.

[31] Wu Y, Mao G, Fan H, Song A, Zhang YP, Chen H (2017). Biochemical properties of GH94 cellodextrin phosphorylase THA_1941 from a thermophilic eubacterium *Thermosipho africanus* TCF52B with cellobiose phosphorylase activity. Sci Rep.

[32] Bulik S, Grimbs S, Huthmacher C, Selbig J, Holzhütter HG (2009). Kinetic hybrid models composed of mechanistic and simplified enzymatic rate laws - a promising method for speeding up the kinetic modelling of complex metabolic networks. FEBS J.

[33] Toya Y, Shimizu H (2013). Flux analysis and metabolomics for systematic metabolic engineering of microorganisms. Biotechnol Adv.

[34] Krishnareddy M, Kim Y-K, Kitaoka M, Mori Y, Hayashi K (2002). Cellodextrin phosphorylase from *Clostridium thermocellum* YM4 strain expressed in *Escherichia coli*. J Appl Glycosci.

[35] Segel I-H (1993). Enzyme kinetics: behavior and analysis of rapid equilibrium and steady-state enzyme systems (Wiley Classics Library).

[36] Gardossi L, Poulsen PB, Ballesteros A, Hult K, Švedas VK, Vasić-Rački Đ, Carrea G, Magnusson A, Schmid A, Wohlgemuth R, Halling PJ (2010). Guidelines for reporting of biocatalytic reactions. Trends Biotechnol.

[37] Flamholz A, Noor E, Bar-Even A, Milo R (2012). eQuilibrator - the biochemical thermodynamics calculator. Nucleic Acids Res.

[38] Ross T (1996). Indices for performance evaluation of predictive models in food microbiology. J Appl Bacteriol.

[39] Saheki S, Takeda A, Shimazu T (1985). Assay of inorganic phosphate in the mild pH range, suitable for measurement of glycogen phosphorylase activity. Anal Biochem.

[40] Hoops S, Sahle S, Gauges R, Lee C, Pahle J, Simus N, Singhal M, Xu L, Mendes P, Kummer U (2006). COPASI—a COmplex PAthway SImulator. Bioinformatics.

[41] Miller JN, Miller Jane C. (2010). Statistics and chemometrics for analytical chemistry.

