# Supplementary material for: Kinetic modeling of phosphorylase-catalyzed iterative β-1,4-glycosylation for degree of polymerization-controlled synthesis of soluble cello-oligosaccharides
Source: Biotechnol Biofuels. 2021 Jun 10;14:134. doi: 10.1186/s13068-021-01982-2 (PMC8194188; doi:10.1186/s13068-021-01982-2)
Supplement: Supplementary file 1 — Additional file 1. Initial rate kinetic analyses of enzyme (CcCdP) towards acceptors (cellobiose or cellotriose) and donor (αGlc1-P): Figure S1 and S2; Semi logarithmic relationship between solubilities of COS and their respective number of glucose molecules: Figure S3; COS precipitation dynamics: Figure S4; Comparison of experimental data from time course analyses with data obtained from model predictions: Figure S5. Results obtained from evaluating model quality: Table S1; Correlation matrix of fitted parameters: Table S2. [file 13068_2021_1982_MOESM1_ESM.docx]

Supplementary Information - Additional file 1

**Kinetic modeling of phosphorylase-catalyzed iterative β-1,4-glycosylation for degree of polymerization-controlled synthesis of soluble cello-oligosaccharides**

Mario KLIMACEK^1^, Chao ZHONG^1^, Bernd NIDETZKY^1,2,*^

^1^Institute of Biotechnology and Biochemical Engineering, Graz University of Technology, NAWI Graz, Graz, Austria

^2^Austrian Centre of Industrial Biotechnology (acib), Graz, Austria

* Corresponding author (B.N.); e-mail: bernd.nidetzky@tugraz.at

M.K. and C.Z. contributed equally.

**Supplementary Figures**


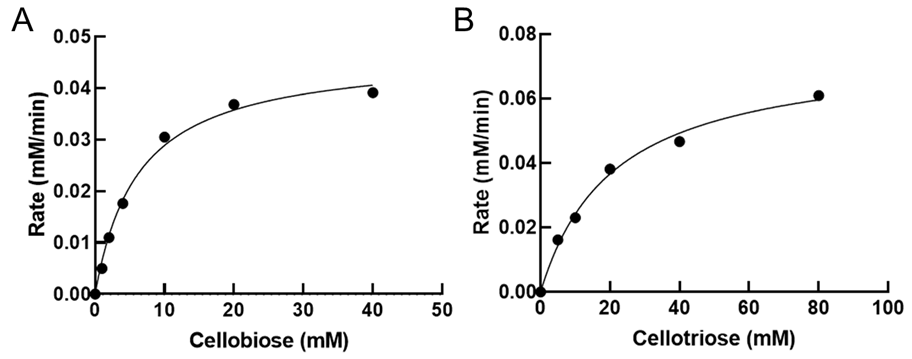


**Figure S1.** **Initial rate kinetic analysis of cellodextrin phosphorylase (from** ***Clostridium cellulosi*, *Cc*CdP) using A) cellobiose and B) cellotriose as the acceptor**. Reaction (50 mM MES, pH 7.0) was done at 45°C and 400 rpm agitation rate on a ThermoMixer C (Eppendorf, Vienna, Austria). Acceptor concentrations (cellobiose 1.0 - 40 mM; cellotriose 5.0 - 80 mM) were varied at saturated αGlc1-*P* concentration (25 mM). The phosphate release within 5 min was measured. Initial rates were plotted against the varied substrate concentration and analyzed under the Michaelis-Menten model with non-linear regression fitting (GraphPad Prism 9). The apparent Michaelis constant (*K*_M_) and the maximal velocity (*V*_max_) were accordingly calculated.


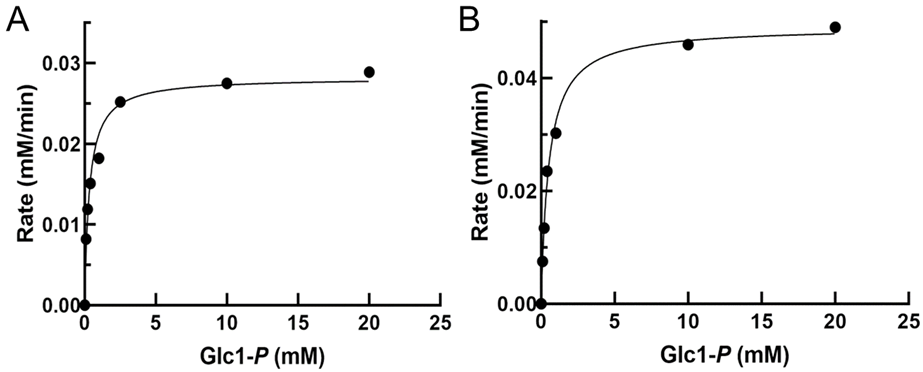


**Figure S2.** **Initial rate kinetic analysis of *Cc*CdP using αGlc1-*P* and cellobiose at A) 5 mM and B) 100 mM**. Reactions (50 mM MES, pH 7.0) were performed under same condition as above (Figure S1). The phosphate release within 5 min was measured. Initial rates were plotted against the varied substrate concentration and analyzed under the Michaelis-Menten model with non-linear regression fitting (GraphPad Prism 9). The *K*_M_ and *V*_max_ were accordingly calculated.


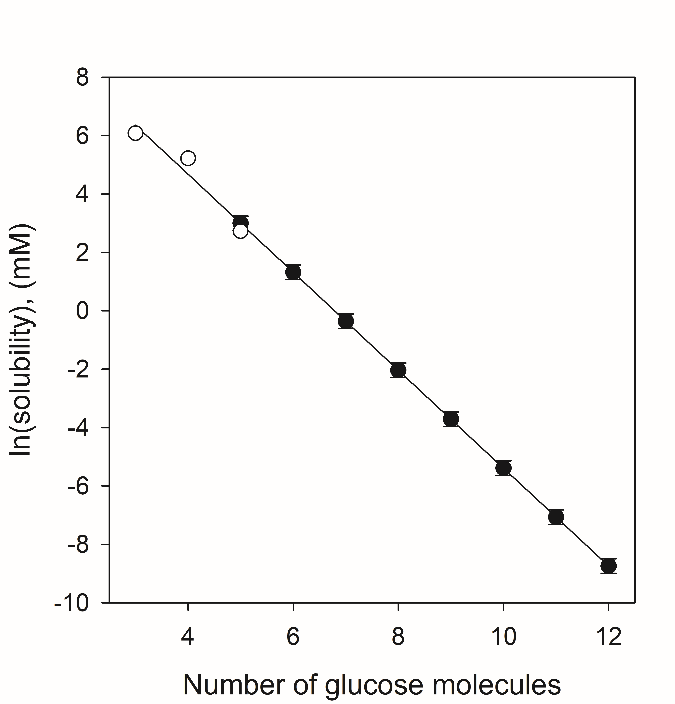


**Figure S3. A semi logarithmic relationship between solubilities of COS in mM and their respective number of glucose molecules was used to estimate the solubility of COS > G5 (black circles).** Solubilities at 45°C were calculated for G3 – G5 (white circles) [1]. Regression parameters from a linear fit of G3 – G5 were used to determine solubility estimates of COSs > G5 (slope = -1.68 ± 0.47; intercept = 11.4 ± 1.9; r^2^ = 0.93). The relative error on the slope value (=0.47 / 1.68 = 0.28) was used to calculate boundaries for G5 – G12 (error bars).


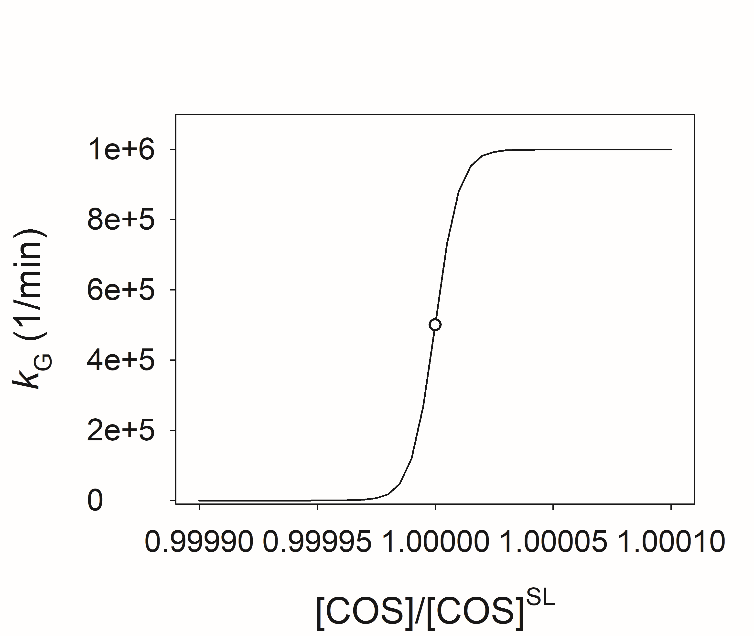


**Figure S4. Precipitation dynamics.** The precipitation rate increases from 0 to 10^6^ min^-1^. The solubility limit [COS]^SL^ is indicated by an empty circle.


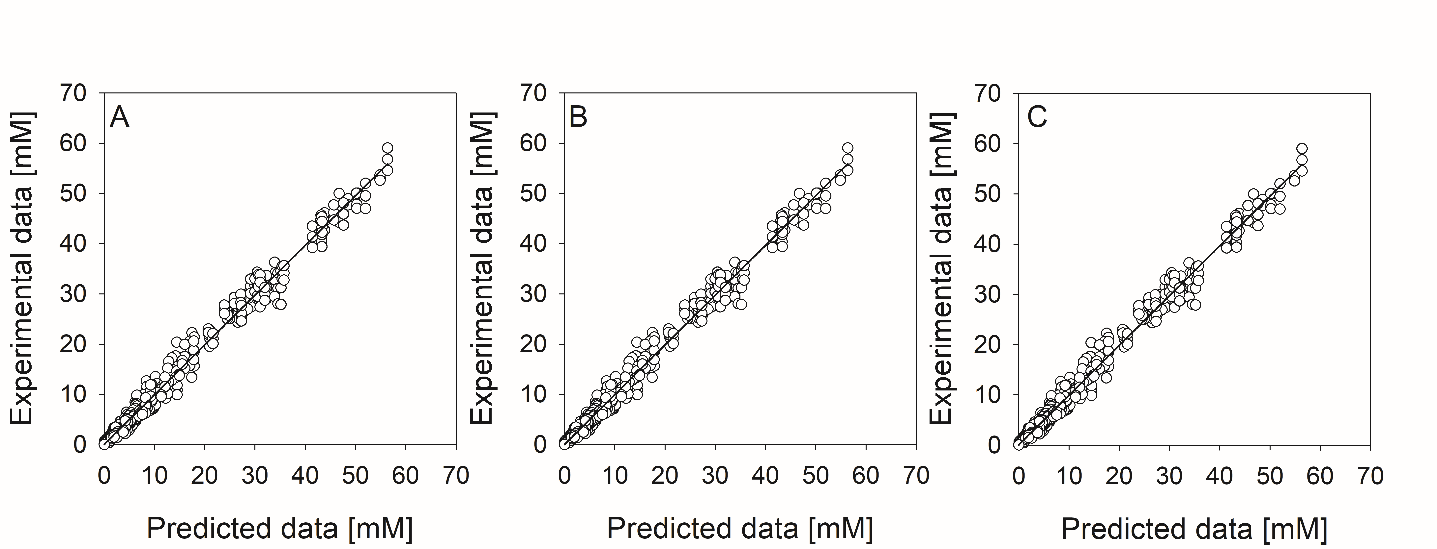


**Figure S5**. **Comparison of experimental data from time course analyses with data obtained from model predictions.** Panels A, B and C show correlations for kinetic models PE18, PE19 and PE20, respectively. Solid lines display resulting fits from linear regression analysis. Almost identical correlation coefficients *R*^2^ (= 0.9906) and slope values (= 0.9906 ± 0.0027) were obtained for the three models.

**Supplementary Tables**

| **Table S1.** Results obtained from evaluating model quality | | | |
| --- | --- | --- | --- |
|  | PE18 | PE19 | PE20 |
| Root-mean square error (RMSE) | 1.3261 | 1.3262 | 1.3230 |
| Bias factor | 1.012 | 1.012 | 1.014 |
| Accuracy factor | 1.141 | 1.141 | 1.140 |

| **Table S2.** Correlation matrix of fitted parameters | | | | | | | | | | | | | | | | | | | |
| --- | --- | --- | --- | --- | --- | --- | --- | --- | --- | --- | --- | --- | --- | --- | --- | --- | --- | --- | --- |
| Parameter estimation | Parameter | *K*_eq_ | *K*_iGlc1P_ | *K*_Glc1P_ | *K*_G2_ | *K*_G3_ | *K*_G4_ | *k*_2_ | *k*_3_ | *k*_4_ | *k*_5_ | [G5]^SL^ | [G6]^SL^ | [G7]^SL^ | [G8]^SL^ | [G9]^SL^ | [G10]^SL^ | [G11]^SL^ | [G12]^SL^ |
| PE18 | *K*_eq_ | 1.00 | -0.01 | 0.00 | 0.36 | 0.18 | 0.10 | 0.03 | -0.03 | -0.02 | -0.03 | -0.03 | 0.02 | 0.15 | -0.19 | 0.03 | 0.07 | -0.04 | -0.01 |
| PE19 |  | 1.00 | -0.02 | -0.01 | -0.13 | -0.15 | -0.17 | -0.17 | -0.18 | -0.18 | -0.18 | 0.03 | -0.03 | 0.26 | -0.14 | -0.08 | 0.02 | -0.11 | 0.21 |
| PE20 |  | 1.00 | -0.03 | -0.01 | -0.09 | -0.11 | -0.13 | -0.12 | -0.15 | -0.15 | -0.15 | 0.03 | -0.03 | 0.24 | -0.05 | -0.04 | 0.13 | 0.05 | 0.01 |
| PE18 | *K*_iGlc1P_ | -0.01 | 1.00 | 0.78 | -0.07 | -0.06 | 0.00 | -0.05 | 0.03 | 0.02 | 0.03 | 0.01 | -0.01 | 0.00 | -0.01 | 0.01 | -0.02 | -0.02 | 0.01 |
| PE19 |  | -0.02 | 1.00 | 0.78 | 0.09 | 0.09 | 0.10 | 0.09 | 0.11 | 0.11 | 0.10 | -0.02 | 0.02 | -0.04 | -0.11 | 0.04 | -0.03 | 0.06 | -0.03 |
| PE20 |  | -0.03 | 1.00 | 0.78 | 0.08 | 0.08 | 0.09 | 0.08 | 0.09 | 0.09 | 0.09 | 0.02 | -0.02 | -0.04 | -0.03 | -0.02 | -0.04 | -0.07 | 0.06 |
| PE18 | *K*_Glc1P_ | 0.00 | 0.78 | 1.00 | -0.11 | -0.08 | -0.01 | -0.07 | 0.02 | 0.02 | 0.02 | 0.00 | 0.00 | 0.01 | -0.02 | 0.01 | -0.01 | -0.02 | 0.00 |
| PE19 |  | -0.01 | 0.78 | 1.00 | 0.10 | 0.10 | 0.12 | 0.10 | 0.12 | 0.12 | 0.12 | -0.03 | 0.02 | -0.02 | -0.16 | 0.04 | -0.03 | 0.06 | -0.02 |
| PE20 |  | -0.01 | 0.78 | 1.00 | 0.08 | 0.08 | 0.09 | 0.07 | 0.09 | 0.09 | 0.09 | 0.03 | -0.03 | -0.03 | -0.02 | -0.03 | -0.04 | -0.09 | 0.08 |
| PE18 | *K*_G2_ | 0.36 | -0.07 | -0.11 | 1.00 | 0.92 | 0.83 | 0.75 | 0.65 | 0.66 | 0.66 | 0.55 | -0.52 | 0.01 | -0.18 | 0.19 | -0.31 | -0.33 | -0.20 |
| PE19 |  | -0.13 | 0.09 | 0.10 | 1.00 | 1.00 | 1.00 | 0.98 | 0.99 | 0.99 | 0.99 | 0.04 | -0.04 | -0.38 | -0.13 | 0.15 | -0.42 | 0.27 | -0.27 |
| PE20 |  | -0.09 | 0.08 | 0.08 | 1.00 | 1.00 | 1.00 | 0.98 | 0.99 | 0.99 | 0.99 | 0.07 | -0.08 | -0.40 | -0.41 | -0.02 | -0.43 | -0.16 | 0.51 |
| PE18 | *K*_G3_ | 0.18 | -0.06 | -0.08 | 0.92 | 1.00 | 0.75 | 0.95 | 0.56 | 0.57 | 0.58 | 0.49 | -0.47 | -0.01 | -0.16 | 0.18 | -0.33 | -0.29 | -0.12 |
| PE19 |  | -0.15 | 0.09 | 0.10 | 1.00 | 1.00 | 0.99 | 0.99 | 0.99 | 0.99 | 0.99 | 0.04 | -0.04 | -0.39 | -0.13 | 0.15 | -0.43 | 0.27 | -0.27 |
| PE20 |  | -0.11 | 0.08 | 0.08 | 1.00 | 1.00 | 0.99 | 0.99 | 0.99 | 0.99 | 0.99 | 0.07 | -0.08 | -0.40 | -0.42 | 0.00 | -0.43 | -0.17 | 0.51 |
| PE18 | *K*_G4_ | 0.10 | 0.00 | -0.01 | 0.83 | 0.75 | 1.00 | 0.58 | 0.96 | 0.96 | 0.94 | 0.77 | -0.72 | -0.01 | -0.17 | 0.21 | -0.35 | -0.46 | -0.37 |
| PE19 |  | -0.17 | 0.10 | 0.12 | 1.00 | 0.99 | 1.00 | 0.98 | 1.00 | 1.00 | 1.00 | 0.04 | -0.04 | -0.39 | -0.13 | 0.13 | -0.41 | 0.27 | -0.28 |
| PE20 |  | -0.13 | 0.09 | 0.09 | 1.00 | 0.99 | 1.00 | 0.98 | 1.00 | 1.00 | 1.00 | 0.07 | -0.08 | -0.41 | -0.40 | -0.03 | -0.43 | -0.16 | 0.50 |
| PE18 | *k*_2_ | 0.03 | -0.05 | -0.07 | 0.75 | 0.95 | 0.58 | 1.00 | 0.40 | 0.42 | 0.43 | 0.38 | -0.36 | -0.02 | -0.13 | 0.15 | -0.30 | -0.22 | -0.03 |
| PE19 |  | -0.17 | 0.09 | 0.10 | 0.98 | 0.99 | 0.98 | 1.00 | 0.97 | 0.97 | 0.97 | 0.04 | -0.04 | -0.39 | -0.13 | 0.16 | -0.44 | 0.26 | -0.27 |
| PE20 |  | -0.12 | 0.08 | 0.07 | 0.98 | 0.99 | 0.98 | 1.00 | 0.97 | 0.97 | 0.97 | 0.06 | -0.07 | -0.39 | -0.43 | 0.01 | -0.42 | -0.18 | 0.52 |
| PE18 | *k*_3_ | -0.03 | 0.03 | 0.02 | 0.65 | 0.56 | 0.96 | 0.40 | 1.00 | 1.00 | 0.97 | 0.78 | -0.73 | -0.02 | -0.15 | 0.20 | -0.32 | -0.46 | -0.42 |
| PE19 |  | -0.18 | 0.11 | 0.12 | 0.99 | 0.99 | 1.00 | 0.97 | 1.00 | 1.00 | 1.00 | 0.04 | -0.04 | -0.39 | -0.12 | 0.13 | -0.40 | 0.27 | -0.28 |
| PE20 |  | -0.15 | 0.09 | 0.09 | 0.99 | 0.99 | 1.00 | 0.97 | 1.00 | 1.00 | 1.00 | 0.07 | -0.08 | -0.42 | -0.39 | -0.03 | -0.43 | -0.15 | 0.49 |
| PE18 | *k*_4_ | -0.02 | 0.02 | 0.02 | 0.66 | 0.57 | 0.96 | 0.42 | 1.00 | 1.00 | 0.97 | 0.78 | -0.73 | -0.02 | -0.14 | 0.20 | -0.33 | -0.46 | -0.42 |
| PE19 |  | -0.18 | 0.11 | 0.12 | 0.99 | 0.99 | 1.00 | 0.97 | 1.00 | 1.00 | 1.00 | 0.04 | -0.04 | -0.39 | -0.12 | 0.13 | -0.40 | 0.27 | -0.28 |
| PE20 |  | -0.15 | 0.09 | 0.09 | 0.99 | 0.99 | 1.00 | 0.97 | 1.00 | 1.00 | 1.00 | 0.07 | -0.08 | -0.42 | -0.39 | -0.03 | -0.43 | -0.15 | 0.49 |
| PE18 | *k*_5_ | -0.03 | 0.03 | 0.02 | 0.66 | 0.58 | 0.94 | 0.43 | 0.97 | 0.97 | 1.00 | 0.76 | -0.70 | 0.03 | -0.02 | 0.23 | -0.31 | -0.46 | -0.44 |
| PE19 |  | -0.18 | 0.10 | 0.12 | 0.99 | 0.99 | 1.00 | 0.97 | 1.00 | 1.00 | 1.00 | 0.04 | -0.03 | -0.39 | -0.12 | 0.13 | -0.40 | 0.28 | -0.28 |
| PE20 |  | -0.15 | 0.09 | 0.09 | 0.99 | 0.99 | 1.00 | 0.97 | 1.00 | 1.00 | 1.00 | 0.07 | -0.07 | -0.41 | -0.37 | -0.03 | -0.44 | -0.15 | 0.49 |

| **Table S2.** (continued) | | | | | | | | | | | | | | | | | | | |
| --- | --- | --- | --- | --- | --- | --- | --- | --- | --- | --- | --- | --- | --- | --- | --- | --- | --- | --- | --- |
| Parameter estimation | Parameter | *K*_eq_ | *K*_iG1P_ | *K*_G1P_ | *K*_G2_ | *K*_G3_ | *K*_G4_ | *k*_2_ | *k*_3_ | *k*_4_ | *k*_5_ | [G5]^SL^ | [G6]^SL^ | [G7]^SL^ | [G8]^SL^ | [G9]^SL^ | [G10]^SL^ | [G11]^SL^ | [G12]^SL^ |
| PE18 | [G5]^SL^ | -0.03 | 0.01 | 0.00 | 0.55 | 0.49 | 0.77 | 0.38 | 0.78 | 0.78 | 0.76 | 1.00 | -0.95 | -0.16 | -0.06 | -0.24 | -0.53 | -0.03 | -0.42 |
| PE19 |  | 0.03 | -0.02 | -0.03 | 0.04 | 0.04 | 0.04 | 0.04 | 0.04 | 0.04 | 0.04 | 1.00 | -0.89 | 0.02 | -0.07 | -0.71 | -0.37 | 0.03 | 0.06 |
| PE20 |  | 0.03 | 0.02 | 0.03 | 0.07 | 0.07 | 0.07 | 0.06 | 0.07 | 0.07 | 0.07 | 1.00 | -0.86 | 0.00 | 0.21 | -0.84 | -0.06 | -0.23 | 0.30 |
| PE18 | [G6]^SL^ | 0.02 | -0.01 | 0.00 | -0.52 | -0.47 | -0.72 | -0.36 | -0.73 | -0.73 | -0.70 | -0.95 | 1.00 | 0.16 | 0.06 | 0.26 | 0.51 | 0.01 | 0.40 |
| PE19 |  | -0.03 | 0.02 | 0.02 | -0.04 | -0.04 | -0.04 | -0.04 | -0.04 | -0.04 | -0.03 | -0.89 | 1.00 | 0.01 | 0.04 | 0.67 | 0.28 | -0.04 | -0.03 |
| PE20 |  | -0.03 | -0.02 | -0.03 | -0.08 | -0.08 | -0.08 | -0.07 | -0.08 | -0.08 | -0.07 | -0.86 | 1.00 | 0.02 | -0.18 | 0.75 | 0.04 | 0.16 | -0.23 |
| PE18 | [G7]^SL^ | 0.15 | 0.00 | 0.01 | 0.01 | -0.01 | -0.01 | -0.02 | -0.02 | -0.02 | 0.03 | -0.16 | 0.16 | 1.00 | -0.46 | 0.10 | 0.15 | -0.60 | 0.10 |
| PE19 |  | 0.26 | -0.04 | -0.02 | -0.38 | -0.39 | -0.39 | -0.39 | -0.39 | -0.39 | -0.39 | 0.02 | 0.01 | 1.00 | -0.59 | -0.12 | -0.06 | -0.66 | 0.72 |
| PE20 |  | 0.24 | -0.04 | -0.03 | -0.40 | -0.40 | -0.41 | -0.39 | -0.42 | -0.42 | -0.41 | 0.00 | 0.02 | 1.00 | -0.10 | 0.04 | 0.34 | -0.01 | -0.09 |
| PE18 | [G8]^SL^ | -0.19 | -0.01 | -0.02 | -0.18 | -0.16 | -0.17 | -0.13 | -0.15 | -0.14 | -0.02 | -0.06 | 0.06 | -0.46 | 1.00 | -0.22 | -0.10 | 0.42 | -0.04 |
| PE19 |  | -0.14 | -0.11 | -0.16 | -0.13 | -0.13 | -0.13 | -0.13 | -0.12 | -0.12 | -0.12 | -0.07 | 0.04 | -0.59 | 1.00 | -0.06 | 0.54 | 0.75 | -0.91 |
| PE20 |  | -0.05 | -0.03 | -0.02 | -0.41 | -0.42 | -0.40 | -0.43 | -0.39 | -0.39 | -0.37 | 0.21 | -0.18 | -0.10 | 1.00 | -0.48 | -0.22 | 0.36 | -0.18 |
| PE18 | [G9]^SL^ | 0.03 | 0.01 | 0.01 | 0.19 | 0.18 | 0.21 | 0.15 | 0.20 | 0.20 | 0.23 | -0.24 | 0.26 | 0.10 | -0.22 | 1.00 | -0.06 | -0.64 | 0.34 |
| PE19 |  | -0.08 | 0.04 | 0.04 | 0.15 | 0.15 | 0.13 | 0.16 | 0.13 | 0.13 | 0.13 | -0.71 | 0.67 | -0.12 | -0.06 | 1.00 | -0.31 | -0.08 | 0.00 |
| PE20 |  | -0.04 | -0.02 | -0.03 | -0.02 | 0.00 | -0.03 | 0.01 | -0.03 | -0.03 | -0.03 | -0.84 | 0.75 | 0.04 | -0.48 | 1.00 | 0.11 | -0.25 | -0.12 |
| PE18 | [G10]^SL^ | 0.07 | -0.02 | -0.01 | -0.31 | -0.33 | -0.35 | -0.30 | -0.32 | -0.33 | -0.31 | -0.53 | 0.51 | 0.15 | -0.10 | -0.06 | 1.00 | 0.08 | -0.52 |
| PE19 |  | 0.02 | -0.03 | -0.03 | -0.42 | -0.43 | -0.41 | -0.44 | -0.40 | -0.40 | -0.40 | -0.37 | 0.28 | -0.06 | 0.54 | -0.31 | 1.00 | 0.28 | -0.37 |
| PE20 |  | 0.13 | -0.04 | -0.04 | -0.43 | -0.43 | -0.43 | -0.42 | -0.43 | -0.43 | -0.44 | -0.06 | 0.04 | 0.34 | -0.22 | 0.11 | 1.00 | -0.38 | -0.47 |
| PE18 | [G11]^SL^ | -0.04 | -0.02 | -0.02 | -0.33 | -0.29 | -0.46 | -0.22 | -0.46 | -0.46 | -0.46 | -0.03 | 0.01 | -0.60 | 0.42 | -0.64 | 0.08 | 1.00 | -0.13 |
| PE19 |  | -0.11 | 0.06 | 0.06 | 0.27 | 0.27 | 0.27 | 0.26 | 0.27 | 0.27 | 0.28 | 0.03 | -0.04 | -0.66 | 0.75 | -0.08 | 0.28 | 1.00 | -0.85 |
| PE20 |  | 0.05 | -0.07 | -0.09 | -0.16 | -0.17 | -0.16 | -0.18 | -0.15 | -0.15 | -0.15 | -0.23 | 0.16 | -0.01 | 0.36 | -0.25 | -0.38 | 1.00 | -0.22 |
| PE18 | [G12]^SL^ | -0.01 | 0.01 | 0.00 | -0.20 | -0.12 | -0.37 | -0.03 | -0.42 | -0.42 | -0.44 | -0.42 | 0.40 | 0.10 | -0.04 | 0.34 | -0.52 | -0.13 | 1.00 |
| PE19 |  | 0.21 | -0.03 | -0.02 | -0.27 | -0.27 | -0.28 | -0.27 | -0.28 | -0.28 | -0.28 | 0.06 | -0.03 | 0.72 | -0.91 | 0.00 | -0.37 | -0.85 | 1.00 |
| PE20 |  | 0.01 | 0.06 | 0.08 | 0.51 | 0.51 | 0.50 | 0.52 | 0.49 | 0.49 | 0.49 | 0.30 | -0.23 | -0.09 | -0.18 | -0.12 | -0.47 | -0.22 | 1.00 |

**Reference**

1. Taylor JB. The water solubilities and heats of solution of short chain cellulosic oligosaccharides. Trans Faraday Soc. 1957; 53:1198-1203.
